# Conditional Perplexity Scoring for Large Language Model−Generated Differential Diagnoses in Case Reports: Preliminary Computational Evaluation

**DOI:** 10.2196/98819

**Published:** 2026-09-09

**Authors:** Takanobu Hirosawa, Yukinori Harada, Ren Kawamura, Taro Shimizu

**Affiliations:** 1 Department of Diagnostic and Generalist Medicine, Dokkyo Medical University, 880 Kitakobayashi, Mibu-cho, Shimotsuga, Tochigi, 321-0293, Japan, 81 282-87-2498

**Keywords:** artificial intelligence, diagnosis, generative artificial intelligence, large language model, natural language processing

## Abstract

**Background:**

Large language models (LLMs) are increasingly used to generate differential diagnoses from clinical narratives. However, LLM-based diagnostic clinical decision support systems still lack a quantitative measure of how strongly a diagnosis is supported by the available case description. Conditional perplexity score quantifies how predictable a target text is given in a preceding context, with lower scores indicating greater predictability. We hypothesized that this concept can be adapted to diagnostic reasoning by treating the prediagnostic case description as the context and a diagnosis as the target text.

**Objective:**

This study aims to evaluate whether conditional perplexity scores, computed by an independent LLM and conditioned on case-report narratives, differ between physician-verified correct and incorrect LLM-generated diagnoses. Specifically, we hypothesized that the correct LLM-generated diagnosis verified by physicians would have lower conditional perplexity scores than incorrect LLM-generated differential diagnoses. A secondary outcome was to compare this scoring behavior across differential diagnosis lists generated by different LLMs.

**Methods:**

We performed a preliminary computational analysis of 392 peer-reviewed diagnostic case reports published in the *American Journal of Case Reports* in 2022. For each case, the prediagnostic clinical description was used as the conditioning context, and the case report–defined final diagnoses were treated as the gold standard. Conditional perplexity scores for differential diagnosis lists previously generated by LLaMA2, Bard, and GPT-4 were computed using an independent longer-context LLM, Qwen2.5‐1.5B. We compared case report–defined final diagnoses, correct LLM-generated diagnoses verified by physicians, and incorrect generated diagnoses using nonparametric comparisons and receiver operating characteristic analyses.

**Results:**

All 392 cases had complete case descriptions and case report–defined final diagnoses. Across the top-10 differential diagnosis lists generated by LLaMA2, Bard, and GPT-4, 823 correct LLM-generated diagnoses verified by physicians and 10,875 incorrect generated diagnoses were analyzed. Case report–defined final diagnoses had lower conditional perplexity scores than incorrect generated diagnoses (median 39.9, IQR 17.7‐119.9 vs median 133.3, IQR 37.5‐672.1). Correct LLM-generated diagnoses also had lower conditional perplexity scores than incorrect LLM-generated diagnoses (median 43.3, IQR 16.6‐147.5 vs median 133.3, IQR 37.6‐672.1). Candidate-level discrimination was moderate overall (area under the receiver operating characteristic curve [AUC] 0.666, 95% CI 0.644‐0.689) and was the highest for GPT-4–generated differential diagnosis lists (AUC 0.678, 95% CI 0.652‐0.705), followed by LLaMA2 (AUC 0.662, 95% CI 0.625‐0.698) and Bard (AUC 0.648, 95% CI 0.617‐0.681). In within-case analyses, correct diagnoses had lower conditional perplexity than the mean incorrect diagnosis in 88.1% (237/269) to 91.1% (195/214) of evaluable lists.

**Conclusions:**

Conditional perplexity provided a moderate quantitative signal associated with physician-verified correctness but did not reliably rank the correct diagnosis ahead of the strongest incorrect candidate, limiting its use as a stand-alone reranking method.

## Introduction

### Diagnostic Excellence and the Challenge of Differential Diagnosis

Accurate diagnosis is a central task in clinical reasoning and a major determinant of patient safety, timely treatment, and efficient access to health care resources [1]. Diagnostic error remains an important cause of preventable harm, and improving diagnostic performance has therefore become a priority in contemporary health care quality and safety efforts [2-8]. In this context, the concept of diagnostic excellence emphasizes not only diagnostic accuracy but also timeliness, communication, equity, patient-centeredness, and appropriate use of available data and expertise during the diagnostic process [9,10]. Tools that help clinicians generate, compare, and revisit diagnostic hypotheses may therefore contribute to diagnostic excellence when they are thoughtfully integrated into real-world clinical workflows [11].

### Diagnostic Clinical Decision Support Systems

Clinical decision support systems (CDSSs) have been proposed as one approach to achieving diagnostic excellence [12,13]. CDSSs are commonly classified as knowledge-based or non–knowledge-based systems. Knowledge-based CDSSs, including rule-based systems and Bayesian models, rely on explicitly encoded clinical knowledge to support clinicians in structuring problem representations and reducing the omission of relevant differential diagnoses. However, many traditional systems require structured inputs, rely on manually curated rules, or show limited flexibility when confronted with the narrative, ambiguous, and evolving nature of real clinical information [14-16]. As electronic clinical text and biomedical knowledge have expanded, interest has grown in computational approaches capable of operating directly on free-text clinical information, physical examination findings, laboratory results, and imaging summaries without manual feature engineering [17,18].

### Large Language Models as Emerging Diagnostic Support Systems

Recent large language models (LLMs) have renewed interest in diagnostic CDSSs because they can process clinical narratives and generate differential diagnoses using natural language processing techniques [19-21]. This capability has prompted interest in their potential role as supportive tools, particularly for hypothesis generation, prioritization of rare diseases, and medical education [22-24]. At the same time, the clinical use of LLMs raises important concerns regarding reliability, calibration, transparency, and explainability [25-27]. Model outputs may appear fluent and convincing even when the underlying reasoning is incomplete, unsupported by the case details, or influenced by spurious textual associations [28,29]. Consequently, established methods are needed not only to assess whether an LLM-generated differential diagnosis list can include the final diagnosis but also to quantify how strongly a differential diagnosis is supported by the preceding clinical narrative.

### Limitations of Current Evaluation Approaches

Many prior evaluations of LLM-based diagnostic performance have focused on whether the final diagnosis appears anywhere in a model-generated list or on its ordinal rank within a differential diagnosis list [30-33]. These measures are clinically intuitive and remain useful for benchmarking diagnostic CDSS performance. To move beyond simple accuracy and evaluate the quality of the reasoning process, recent studies have increasingly adopted clinical reasoning rubrics such as the Revised-IDEA (R-IDEA) score. The R-IDEA framework uses a 10-point scale to grade 4 core domains of clinical reasoning: the interpretive summary, the differential diagnosis, the explanation of the leading diagnosis, and the justification for alternative diagnoses [34].

However, these approaches do not directly quantify the degree of compatibility between narrative clinical information and a specific diagnosis. A diagnosis could be placed in a top-*k* list, meaning it is among the first *k* differential diagnoses proposed by a model, while still being only weakly supported by the available clinical information [35]. In contrast, a diagnosis that is highly consistent with the narrative clinical information might receive stronger model-based support even if it was generated by a different model or phrased differently. A complementary quantitative framework is therefore needed to evaluate narrative-to-diagnosis compatibility directly.

### Explainable AI and the Need for Quantitative Compatibility Metrics

Such a framework is particularly relevant for the development of explainable artificial intelligence (XAI) in medicine [36,37]. In high-stakes clinical settings, explainability extends beyond producing plausible reasoning; it also involves providing transparent, reproducible signals that help users understand why one diagnostic hypothesis is more strongly supported than another. From this perspective, a useful CDSS metric should be externally computable, comparable across differential diagnosis lists, and interpretable as a function of the observed case narrative rather than an internal score produced by a single LLM. The quantitative measures may therefore help make LLM-assisted differential diagnosis more inspectable and auditable [38].

### LLM-Based Conditional Perplexity Score as a Compatibility Measure

Perplexity score is a standard NLP metric used to evaluate how well an LLM predicts a sequence of tokens [39,40]. A token is the basic unit of data that an LLM processes and generates. In an LLM, the conditional perplexity score of a target sequence can be estimated token by token, given the preceding text as context [41]. Applied to clinical diagnosis, a lower conditional negative log-likelihood (NLL) and a correspondingly lower conditional perplexity score indicate that a specific diagnosis string is more predictable to the scoring model after it has processed the case narrative. In this context, the conditional perplexity score can be repurposed as a reproducible measure of text-based compatibility between the available clinical narrative and a proposed differential diagnosis.

This approach offers several potential advantages for both CDSS research and the evaluation of XAI in medicine. First, it provides a continuous score rather than binary correct or incorrect judgments or rubric-based scores. Second, it facilitates a direct comparison among multiple differential diagnoses generated by different LLMs. Third, because the score is conditioned explicitly on the prediagnostic narrative, it offers a transparent way to assess whether corrected LLM-generated diagnoses, verified by physicians, exhibit higher text-based compatibility than incorrect differential diagnoses. Fourth, such scores may serve as a reranking signal within ensemble CDSS approaches [42], in which differential diagnoses generated by multiple models can be aggregated, scored for narrative compatibility, and reordered to surface the most plausible clinical conclusion.

### Remaining Knowledge Gaps

Despite growing interest in LLM-assisted diagnostic reasoning, several important questions remain insufficiently addressed in the existing literature. First, it is unclear whether LLM-based compatibility metrics can reliably distinguish correct LLM-generated diagnoses verified by physicians from incorrect LLM-generated differential diagnoses when conditioned on the same case descriptions. Second, prior work has rarely examined whether externally computed LLM-based scores align with correct LLM-generated diagnoses verified by physicians within prediagnostic case descriptions. Third, the extent to which such scores could function as a reproducible reranking signal across differential diagnosis lists generated by different LLM families has not been systematically evaluated. Addressing these gaps is important for determining whether LLM-based metrics can contribute to explainable evaluation and safer clinical decision support.

### Study Objective and Research Questions

In the present study, we evaluated 392 diagnostic case reports published in 2022 in the *American Journal of Case Reports*. Using precompiled differential diagnosis lists generated by LLaMA2, Bard, and GPT-4, we applied a longer-context causal language model as an external scorer to compute conditional perplexity scores for the gold-standard final diagnoses and differential diagnosis lists.

Our primary objective was to determine whether external LLM-based conditional perplexity scores could serve as a quantitative framework for ranking physician-verified correct LLM-generated diagnoses within differential diagnosis lists. The specified aim was to test whether correct diagnoses would receive lower scores than incorrect candidate diagnoses when conditioned on the same prediagnostic case narrative. We further aimed to determine whether this signal remained observable across multiple source LLMs while explicitly treating the score as a text-level compatibility measure rather than a stand-alone measure of clinical reasoning.

1. Compatibility: Do case report–defined final diagnoses receive lower conditional perplexity scores than incorrect LLM-generated differential diagnoses when conditioned on the same prediagnostic case narrative?
2. Candidate discrimination: Within LLM-generated differential diagnosis lists, can a conditional perplexity score distinguish physician-verified correct LLM-generated diagnoses from incorrect differential diagnoses?
3. Cross-model behavior: Does this compatibility signal remain observable across differential diagnosis lists generated by multiple LLMs?

By framing diagnostic evaluation through an LLM-based conditional perplexity score, this study explores a preliminary approach for connecting LLM-based differential diagnosis generation with reproducible evaluation, interpretable decision support, and the broader goal of diagnostic excellence.

## Methods

### Study Setting

**Figure 1.**
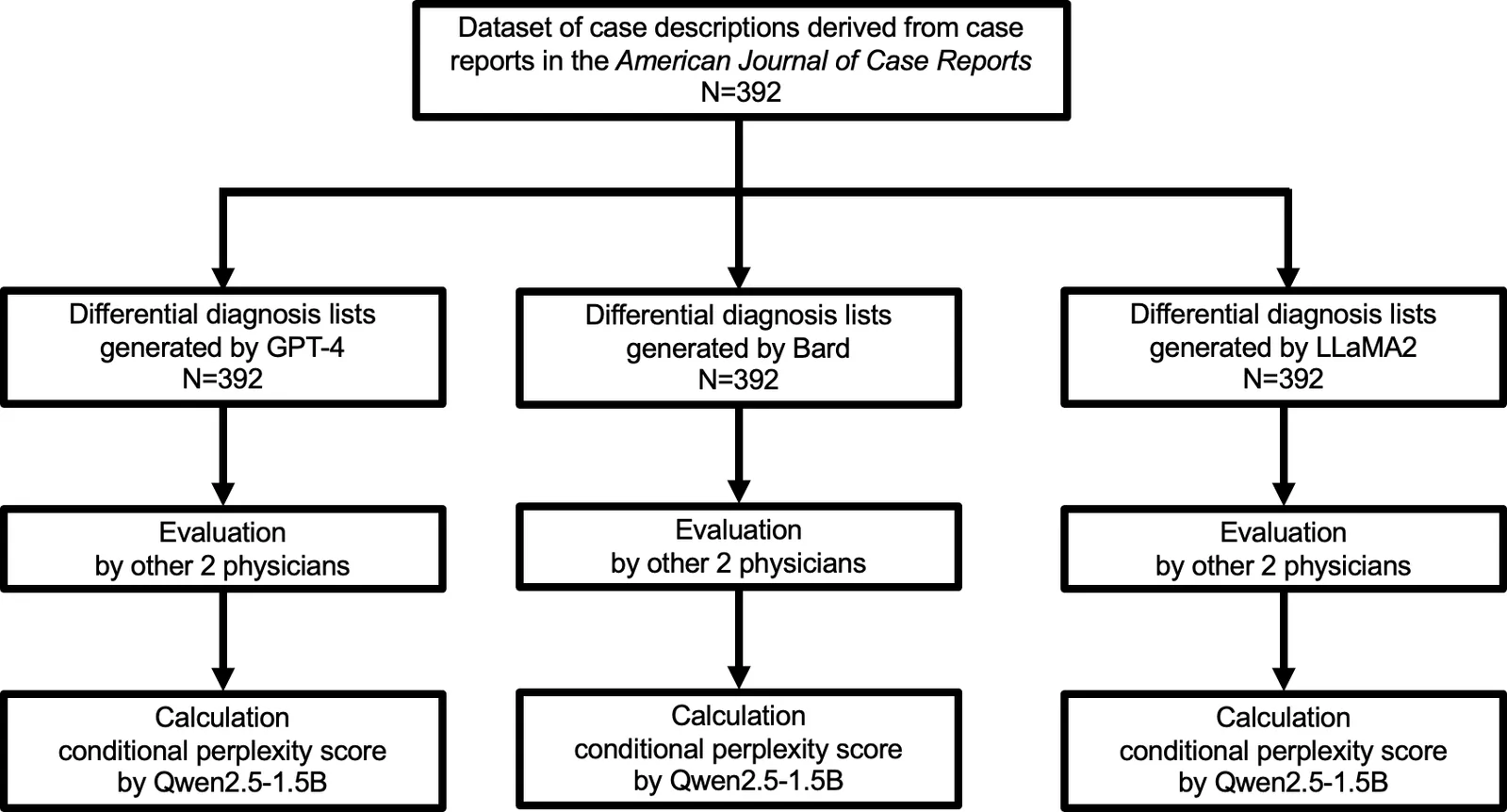
Study workflow. Case reports published in 2022 in the *American Journal of Case Reports* were collected, prediagnostic case descriptions were extracted, case report–defined final diagnoses were recorded, and large language model (LLM)–generated differential diagnosis lists from GPT-4, Bard, and LLaMA2 were rescored by a longer-context LLM to compute conditional perplexity scores.

This was a retrospective computational evaluation of 392 diagnostic case reports published in 2022 in the *American Journal of Case Reports*. The objective was not to generate new diagnoses but to quantify how compatible each diagnosis was with the preceding clinical narrative using a fixed external LLM. The overall study flow is shown in Figure 1.

### Ethical Considerations

This study was a secondary computational analysis of previously published, publicly available case reports and previously generated differential diagnosis lists. The investigators had no contact with patients and did not access medical records, direct identifiers, or nonpublic health information. Because the study used only previously published, publicly available information and did not involve identifiable private information or direct interaction with human participants, additional ethics committee review and informed consent were not sought. Therefore, additional ethics committee review and informed consent were not sought. The source case reports were accessed under the publication and licensing terms of th*e American Journal of Case Reports* and were used for noncommercial computational research. The present study did not redistribute modified full-text case reports. This retrospective computational analysis was not preregistered.

### Data Preprocessing

Clinical information was extracted from the original case reports prior to the final diagnosis. The prediagnostic case descriptions were prepared by the main investigator (TH) after removing the assessment and differential diagnoses. Typical prediagnostic case descriptions included the background, chief concerns, history of present illness, past medical history, physical examination findings, and the results of investigations. This process was validated by another investigator (YH).

For each case, the case report–defined final diagnosis was treated as the gold standard. Duplicate diagnoses were removed after lowercasing while preserving the original order. Physician-verification labels for the LLM-generated differential diagnoses were imported from the source dataset publication. In that source dataset, 2 general internal medicine expert physicians independently reviewed each case report–defined final diagnosis and each AI-generated differential diagnosis list. Each candidate was coded as correct if it accurately matched the final diagnosis with acceptable specificity or was sufficiently close such that appropriate treatment would be initiated without compromising patient safety; candidates judged substantially different from the final diagnosis were coded as incorrect. Disagreements were resolved through consultation with a third general internal medicine expert physician, reviewers were blinded to the AI system that produced each list, and interrater agreement was reported as 88.9% with a Cohen κ coefficient of 0.76 [43]. In the present analysis, physician-verified correctness was based on clinical concordance rather than exact string matching; therefore, clinically equivalent synonyms or differences in diagnostic granularity could be judged correct.

For each case narrative, a standardized prompt was constructed as follows: “Clinical information from a case report: [Insert pre-diagnostic case description]. The final diagnosis is.” The prompt was included in the conditioning context, allowing the analysis to estimate the conditional likelihood of the diagnosis tokens alone.

### Source Differential Diagnosis Lists and Model Provenance

The differential diagnosis lists were generated in a previously published Digital Health study using public web-based chat interfaces [43]. The GPT-4 differential diagnosis lists were generated through the ChatGPT web interface from June 22 to June 29, 2023, using the March 24 version in the default chat mode; custom instructions were not used, and chat history was disabled in the browser used for the study. The Bard differential diagnosis lists were generated through the Google Bard web interface, now Google Gemini, from July 27 to August 1, 2023, before the system transition to Google Gemini; no specific version number or adjustable decoding settings were available, and Bard or Gemini activity was disabled. The LLaMA2 differential diagnosis lists were generated through the public LLaMA2 chatbot web interface from August 3 to August 8, 2023, using the 70B version of the LLaMA2 model by Meta AI; the web interface displayed a temperature of 2.49, top P 0.50, a maximum sequence length of 2048, and default prompt settings. The present study did not regenerate these differential diagnosis lists but rescored the fixed lists with Qwen2.5‐1.5B.

### Scoring an LLM

An LLM with an extended context length, Qwen2.5‐1.5B, was used as the scoring model. Specifically, we used the base checkpoint available from the Hugging Face repository Qwen or Qwen2.5‐1.5B. Because Qwen2.5 supports longer context windows than many earlier open-weight models, it was suitable for long case descriptions [44]. Across all main analytic prompt-diagnosis sequences, all sequences fit within the 32,768-token context window; therefore, sliding-window scoring was not required.

The tokenizer was loaded from the same Hugging Face repository using AutoTokenizer with the fast tokenizer enabled (use_fast=True), and both the conditioning prompt and target diagnosis were tokenized with special-token insertion disabled (add_special_tokens=False). The model was loaded with the Hugging Face transformers library, and inference was performed on graphics processing unit (GPU) or central processing unit (CPU) depending on hardware availability [45]. Because perplexity scores are model-dependent, the use of one general-purpose scorer was treated as a source of uncertainty rather than as evidence that the observed signal would necessarily reproduce across other scorer architectures or biomedical-domain models.

### Conditional Likelihood and Perplexity Calculation

For a clinical context string (*c*) and a candidate diagnosis string (*d*=*x*_1:N_), where (*x*_*i*_) denotes the (*i*)-th diagnosis token and (*N*) denotes the number of diagnosis tokens after tokenization by the scoring model, the conditional probability assigned to the diagnosis by a causal language model with parameters (theta) is defined as follows:


$$P_{\theta }(d|c)=\prod_{i=1}^{N}P_{\theta }(x_{i}|c,x_{§lt;i})$$

The token-level average NLL for the diagnosis, given the clinical context was calculated as:


$$\mathrm{avgNLL}(d|c)=-\frac{1}{N}\sum_{i=1}^{N}\log P_{\theta }(x_{i}|c,x_{§lt;i})$$

The conditional perplexity score was then defined as the exponential of the average negative log-likelihood:


$$\begin{array}{rl} PPL(d|c) & =\exp avgNLL(d|c)\\ =\exp\left(-\frac{1}{N}\sum_{i=1}^{N}\log P_{\theta }(x_{i}|c,x_{§lt;i})\right) \end{array}$$

A lower average NLL and a lower conditional perplexity score indicate that the scoring model considered the diagnosis string more predictable after inputting the case description. When the concatenated prompt and diagnosis fit within the model context window, the entire sequence was scored in a single forward pass, with labels for prompt tokens masked out [46].

### Candidate Ranking Procedure

For each case and each LLM, the 10 differential diagnoses were scored independently using the same conditioned prompt derived from prediagnostic clinical descriptions. Differential diagnoses were then sorted in ascending order of the average NLL. The diagnosis with the lowest average NLL was considered the top-ranked diagnosis according to the scoring model.

### Outcomes

The primary outcome was whether clinically supported diagnoses received lower conditional perplexity than unsupported diagnoses. Clinically supported diagnoses were analyzed as (1) the case report–defined final diagnosis (gold standard) and (2) correct LLM-generated diagnoses verified by physicians, present within LLaMA2-generated, Bard-generated, or GPT-4–generated differential diagnosis lists. The incorrect generated differential diagnoses served as the comparison group. Because conditional perplexity can be affected by lexical frequency, diagnosis-string length, overlap between diagnosis terms and the narrative, and scorer-model familiarity, lower scores were interpreted as greater conditional predictability under the scoring model rather than as direct evidence of case-specific clinical compatibility or clinical reasoning.

Secondary outcomes included the discriminative performance of the conditional perplexity score at the candidate level, quantified by the area under the receiver operating characteristic curve (AUC). Additional secondary outcomes included comparisons of conditional perplexity scoring behavior across LLaMA2, Bard, and GPT-4 and within-case paired evaluations. Within cases containing a physician-verified correct diagnosis, the conditional perplexity of the correct diagnosis was compared with the mean, median, and lowest conditional perplexity among incorrect diagnoses from the same differential diagnosis list. The lowest-perplexity incorrect diagnosis represented the most stringent within-list comparator.

### Statistical Analysis

Continuous variables are summarized as medians with IQRs. Receiver operating characteristic (ROC) analyses were performed at the candidate level to assess how well conditional perplexity discriminated physician-verified correct LLM-generated diagnoses from incorrect LLM-generated diagnoses. Because multiple candidate diagnoses were nested within cases, 95% CIs for the area under the ROC curve (AUC) were estimated using case-cluster nonparametric bootstrap resampling, in which cases were sampled with replacement, and all candidate diagnoses belonging to each sampled case were retained [47]. Thus, the primary inferential emphasis was placed on case-cluster bootstrap estimates of candidate-level AUCs and within-case paired comparisons. Within-case comparisons of conditional perplexity scores were performed using 2-sided Wilcoxon signed-rank tests. Ninety-five percent CIs for medians reported in the within-case analyses were estimated using a nonparametric percentile bootstrap with 2000 resamples. All tests were 2-sided, and *P*<.05 was considered statistically significant.

Post hoc sensitivity analyses were performed to address reviewer concerns about lexical confounding and clustering. We derived lexical features for each candidate diagnosis, including diagnosis-string token count, character count, a candidate-corpus token-frequency proxy (mean log token document frequency), token-overlap fraction between the diagnosis string and prediagnostic case description, any token overlap, exact phrase occurrence in the case description, and case description word count. We compared conditional perplexity with lexical-overlap baselines using case-level cluster bootstrap resampling by case. We also fitted an exploratory logistic regression model predicting physician-verified correctness from average NLL, diagnosis length, token-frequency proxy, lexical-overlap fraction, exact phrase occurrence, case length, and source LLM, with SEs clustered by case. These analyses were exploratory because they used a corpus-derived lexical-frequency proxy rather than the scorer model’s true pretraining-token frequency and did not include rescoring against mismatched case descriptions.

Analyses were performed in Python (version 3.12.3). Sample Python code to calculate conditional perplexity scores and the computational environment are shown in Multimedia Appendix 1. Additionally, sample Python code for statistical analysis and post hoc sensitivity analyses, including lexical-overlap features, adjusted logistic regression, case-level cluster-bootstrap AUCs, and within-case comparator analyses, is shown in Multimedia Appendix 2.

## Results

### Dataset Characteristics and Diagnostic Coverage

The final analysis computed conditional perplexity scores for all 392 case report–defined final diagnoses and for 11,698 generated differential diagnoses across the 3 source LLMs, including LLaMA2, Bard, and GPT-4. Overall, the generated lists contained 823 correct LLM-generated diagnoses verified by physicians and 10,875 incorrect differential diagnoses.

Correct diagnosis coverage differed across source models. Of the total 392 cases, LLaMA2 included a correct diagnosis in 214 (54.6%) of the cases, Bard in 269 (68.6%) of the cases, and GPT-4 in 340 (86.7%) of the cases. GPT-4 also achieved the best original-list top-1 coverage (n=214, 54.6%), followed by Bard (n=123, 31.4%) and LLaMA2 (n=90, 23.0%; Table 1).

**Table 1.** Diagnostic coverage of correct large language model (LLM)–generated diagnoses verified by physicians within differential diagnosis lists.

Model	Top-10, n (%)	Top-1, n (%)
LLaMA2	214 (54.6)	90 (23.0)
Bard	269 (68.6)	123 (31.4)
GPT-4	340 (86.7)	214 (54.6)

In Table 2, we selected 2 representative examples from the dataset: one in which the physician-verified final diagnosis exactly matched the correct LLM-generated diagnosis string, and one in which the correct LLM-generated diagnosis was clinically concordant but not text-identical to the case report–defined diagnosis. In both examples, lower conditional perplexity indicates greater compatibility between the prediagnostic case narrative and the candidate diagnosis.

**Table 2.** Representative examples with conditional perplexity score.

Case title	Model	Case report–defined final diagnosis as gold standard (conditional perplexity score)	Correct LLM^a^-generated diagnosis (conditional perplexity score)	Incorrect LLM-generated diagnosis (conditional perplexity score)^b^
The many faces of immune checkpoint inhibitor-associated pneumonitis: 4 case reports	Bard	Immune checkpoint inhibitor-associated pneumonitis (19.0)	Immune checkpoint inhibitor-associated pneumonitis (19.0)	Infection (30.7)
Solitary soft-tissue metastasis of a pancreatic adenocarcinoma 2 years after curative resection	GPT-4	Soft tissue metastasis from pancreatic adenocarcinoma (12.2)	Metastatic pancreatic adenocarcinoma (9.0)	Fibroma or myofibroma (148.5)

a LLM: large language model.

b Incorrect large language model–generated diagnosis was selected based on the lowest conditional perplexity score within the differential diagnosis list.

### Overall Separation of Clinically Supported Diagnoses From Incorrect Diagnoses

**Figure 2.**
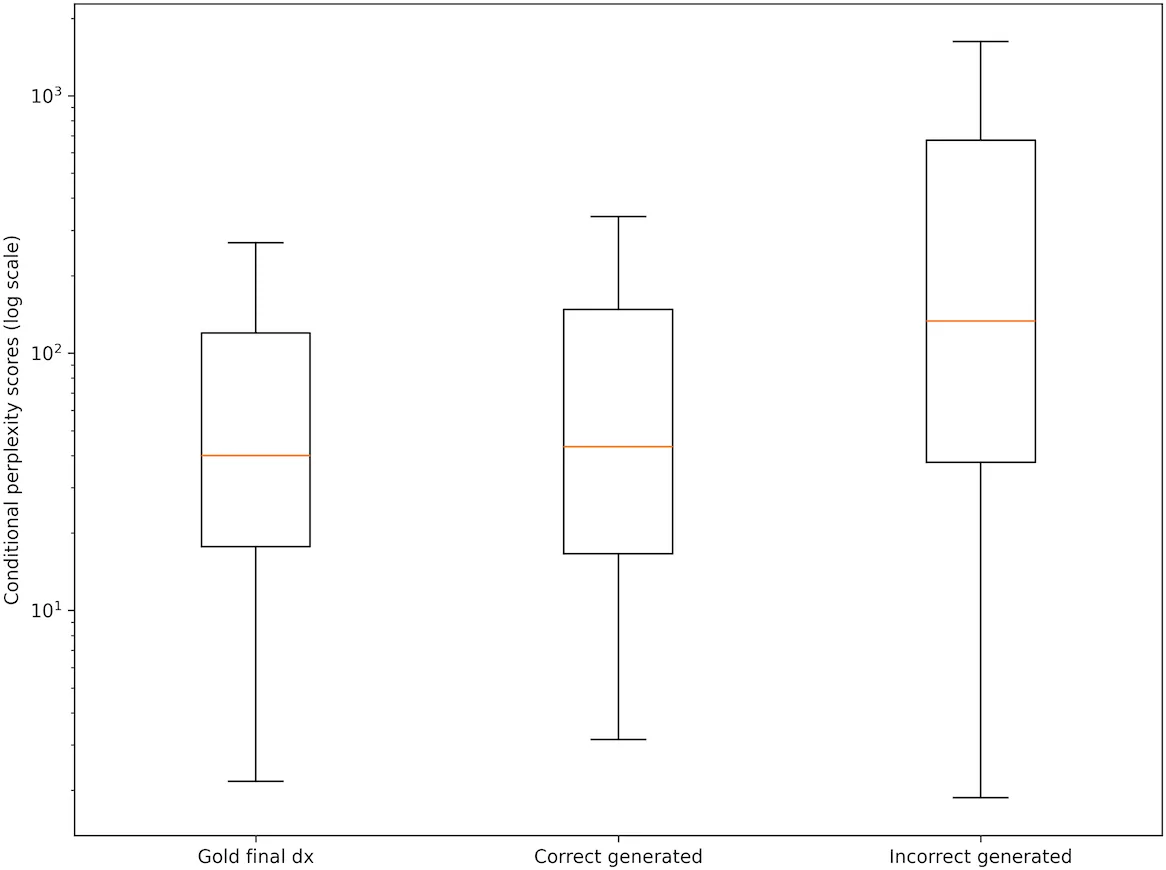
Conditional perplexity distributions show pooled score distributions for case report–defined final diagnoses (gold final dx), correct large language model (LLM)–generated diagnoses verified by physicians (correct generated), and incorrect LLM-generated differential diagnoses (incorrect generated) on a logarithmic *y*-axis.

Case report–defined final diagnoses had substantially lower conditional perplexity scores than incorrect LLM-generated differential diagnoses (median 39.9, IQR 17.7‐119.9 vs median 133.3, IQR 37.6‐672.1; *P*<.001). Correct LLM-generated diagnoses verified by physicians showed a similar pattern, with lower conditional perplexity score than incorrect LLM-generated differential diagnoses overall (median 43.3, IQR 16.6‐147.5; *P*<.001). The distributions of conditional perplexity scores across diagnosis categories are shown descriptively in Figure 2.

Using case-cluster bootstrap resampling to account for candidate diagnoses nested within cases, the pooled AUC for distinguishing physician-verified correct from incorrect generated diagnoses was 0.666 (95% CI 0.644‐0.689; Multimedia Appendix 3). This AUC should be interpreted as exploratory discrimination within a curated case-report dataset rather than as a validated clinical decision threshold (Table 3).

**Table 3.** Conditional perplexity scores according to diagnosis category.

Group	Diagnostic case reports, n	Median conditional perplexity score (IQR)	AUC^a^ vs incorrect (case-cluster bootstrap 95% CI)^b^
Case report–defined final diagnosis	392	39.9 (17.7‐119.9)	—^c^
Correct LLM^d^-generated diagnosis verified by physicians (pooled)	823	43.3 (16.6‐147.5)	0.666 (0.644‐0.689)
Incorrect generated diagnosis (pooled)	10,875	133.3 (37.6‐672.1)	Reference
LLaMA2: correct generated diagnosis	214	45.2 (17.8‐167.1)	0.662 (0.625‐0.698)
LLaMA2: incorrect generated diagnosis	3682	131.3 (38.0‐674.1)	Reference
Bard: correct generated diagnosis	269	55.9 (25.8‐215.3)	0.648 (0.617‐0.681)
Bard: incorrect generated diagnosis	3630	174.9 (48.9‐985.1)	Reference
GPT-4: correct generated diagnosis	340	30.7 (11.5‐91.8)	0.678 (0.652‐0.705)
GPT-4: incorrect generated diagnosis	3563	102.9 (29.0‐462.2)	Reference

a AUC: area under the receiver operating characteristic curve.

b AUCs quantify candidate-level discrimination; 95% CIs were estimated by bootstrap resampling at the case level, retaining all candidate diagnoses within each sampled case.

c Not applicable.

d LLM: large language model.

### Post Hoc Lexical-Overlap and Cluster-Bootstrap Sensitivity Analyses

Post hoc sensitivity analyses are summarized in Multimedia Appendix 3. Lexical overlap alone showed weaker discrimination than conditional perplexity: the pooled AUC was 0.570 (95% CI 0.545‐0.596) for lexical-overlap fraction, 0.567 (95% CI 0.542‐0.589) for any lexical overlap, and 0.500 (95% CI 0.485‐0.515) for exact phrase occurrence in the case description. In the adjusted logistic regression model with case-clustered SEs, lower average NLL, represented as higher-average NLL, remained associated with physician-verified correctness after adjustment for diagnosis length, token-frequency proxy, lexical overlap, exact phrase occurrence, case length, and source LLM (odds ratio 1.36 per 1-unit increase in average NLL, 95% CI 1.27‐1.46; *P*<.001). The combined conditional-perplexity plus lexical-feature model had a cluster-bootstrap AUC of 0.694 (95% CI 0.672‐0.716), compared with 0.639 (95% CI 0.618‐0.662) for the lexical-only model and 0.671 (95% CI 0.649‐0.694) for the conditional-perplexity-only model.

### Model-Specific Scoring Behavior

**Figure 3.**
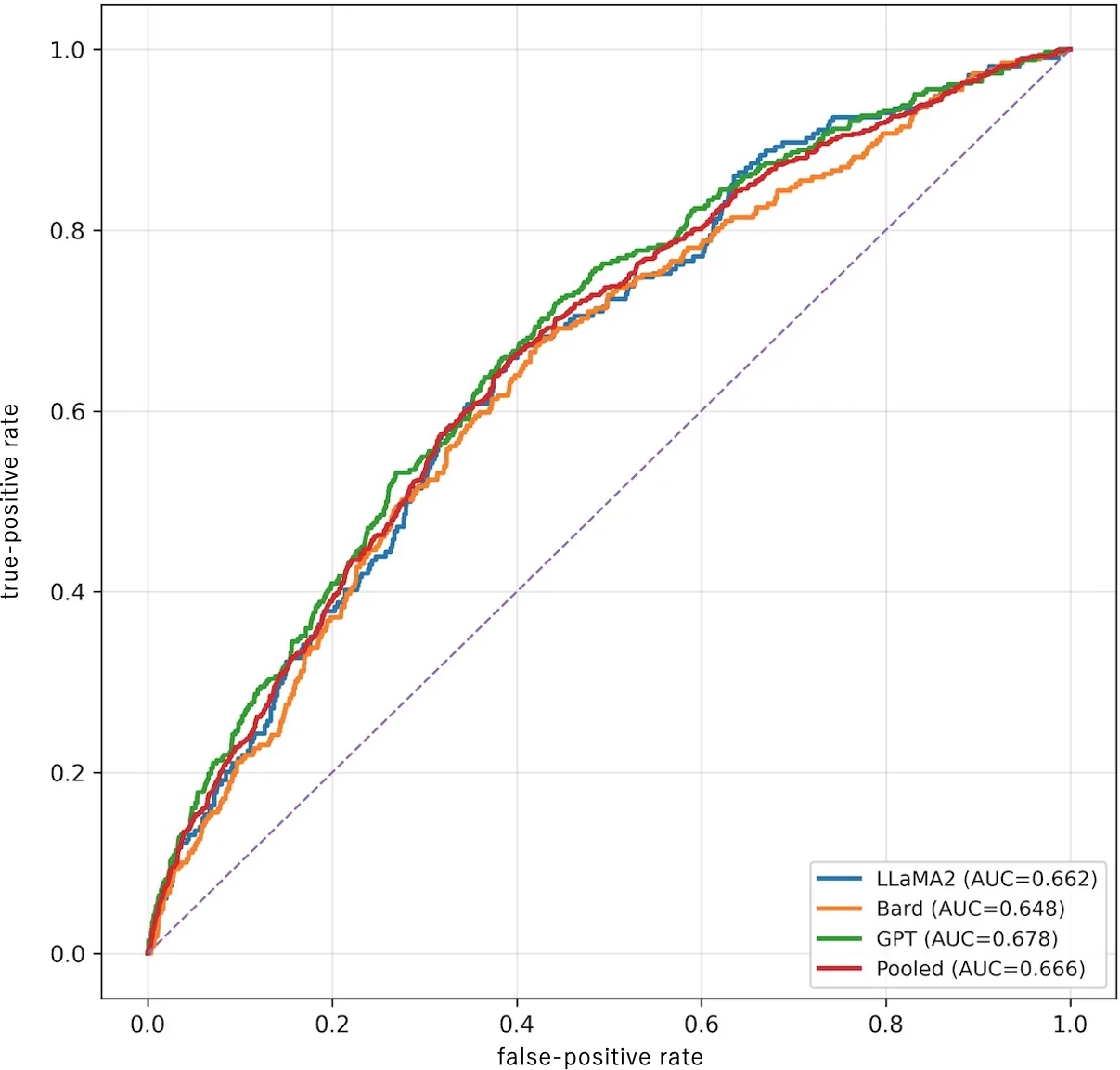
Candidate-level receiver operating characteristic curves for conditional perplexity scores, shown for each source large language model (LLM) and the pooled candidate set. Because candidate diagnoses were nested within cases, uncertainty around area under the receiver operating characteristic curve (AUC) estimates was quantified using case-cluster bootstrap resampling.

Within each LLM as shown in Figure 3, physician-verified correct LLM-generated diagnoses had a lower median conditional perplexity score than incorrect LLM-generated differential diagnoses: LLaMA2 (45.2 vs 131.3), Bard (55.9 vs 174.9), and GPT-4 (30.7 vs 102.9). Candidate-level discrimination was the highest for GPT-4 (AUC 0.678, 95% CI 0.647‐0.707), followed by LLaMA2 (AUC 0.662, 95% CI 0.626‐0.696) and Bard (AUC 0.648, 95% CI 0.616‐0.682).

Correct GPT-4–generated diagnoses had lower conditional perplexity score than those from LLaMA2 and Bard, whereas the difference between correct diagnoses generated by LLaMA2 and Bard was not statistically significant. False-positive conditional perplexity scores also differed across LLMs (*P*<.001), with Bard showing the highest median values and GPT-4 showing the lowest median values.

### Within-Case Paired Comparisons

In paired case-level analyses restricted to cases in which a correct LLM-generated diagnosis verified by physicians was present, the correct diagnosis had a lower conditional perplexity score than the average incorrect diagnosis in 195 out of 214 (91.1%) LLaMA2 cases, 304 out of 340 (89.4%) GPT-4 cases, and 237 out of 269 (88.1%) Bard cases (all Wilcoxon *P*<.001; Table 4). This comparison summarizes separation from the average incorrect candidate and should not be interpreted as indicating that the correct diagnosis was the lowest-perplexity candidate within a list.

**Table 4.** Within-case comparison of correct generated diagnoses versus the mean conditional perplexity of incorrect generated diagnoses.^a^

Source	Paired cases, n	Correct diagnosis median conditional perplexity score (IQR; 95% CI)	Average incorrect diagnosis median conditional perplexity score (IQR; 95% CI)	Correct < average incorrect, n (%)	*P* value^b^
Case report–defined final diagnoses	—^c^	39.9 (17.7-119.9; 33.9‐43.9)	—	—	—
LLaMA2	214	45.1 (17.8-167.1; 36.3‐52.5)	973.5 (292.2-3980.3; 774.3‐1237.3)	195 (91.1)	<.001
Bard	269	55.9 (25.8-215.3; 44.8‐74.7)	1600.3 (443.3-6648.5; 1320.9‐2067.0)	237 (88.1)	<.001
GPT-4	340	30.7 (11.5-91.7; 26.7‐39.1)	692.2 (243.8-2073.8; 555.2‐839.3)	304 (89.4)	<.001

a Median 95% CIs were estimated using a nonparametric percentile bootstrap with 2000 resamples.

b *P* values are derived from 2-sided Wilcoxon signed-rank tests.

c Not applicable.

More stringent within-case analyses showed that the physician-verified correct diagnosis had lower conditional perplexity than the lowest-perplexity incorrect diagnosis in 54 of 214 (25.2%) LLaMA2 lists, 64 of 269 (23.8%) Bard lists, and 82 of 340 (24.1%) GPT-4 lists. Compared with the median incorrect diagnosis, the corresponding proportions were 70.1% (150/214) for LLaMA2, 68.0% (183/269) for Bard, and 74.1% (252/340) for GPT-4. These findings indicate that although correct diagnoses tended to have lower perplexity than the overall distribution of incorrect diagnoses, conditional perplexity did not reliably assign the lowest score to the correct diagnosis within individual lists (Multimedia Appendix 3).

Compared with the case report–defined final diagnosis, the corresponding correct LLM-generated diagnosis verified by physicians had a significantly higher conditional perplexity score for Bard (paired median 39.9 vs 55.9; 2-sided Wilcoxon *P*<.001). However, for both LLaMA2 and GPT-4, the conditional perplexity scores for the correct generated diagnoses were comparable to the case report–defined final diagnoses (LLaMA2 paired median 39.9, IQR 17.7-119.9 vs 45.1, IQR 17.8-167.1; *P*=.07; GPT-4 paired median 39.9, IQR 17.7-119.9 vs 30.7, IQR 11.5-91.7; *P*=.17).

## Discussion

### Principal Findings

In this preliminary evaluation of 392 published case reports, both the case report–defined final diagnosis and the correct LLM-generated diagnosis verified by physicians had lower conditional perplexity scores than incorrect generated diagnoses. These findings indicate that physician-verified correctness was associated with greater conditional predictability under the independent scoring model; however, the present design does not establish case-specific clinical compatibility.

The within-case analyses also clarify the potential role of conditional perplexity in ranking differential diagnoses. Although the physician-verified correct diagnosis had lower conditional perplexity than the mean incorrect diagnosis in approximately 88%‐91% of evaluable lists, the mean is a permissive comparator because incorrect-diagnosis perplexity distributions were highly right-skewed. Against the more stringent lowest-perplexity incorrect candidate, the correct diagnosis had the lower score in only approximately one-quarter of evaluable lists across the 3 source models. Thus, conditional perplexity showed a distribution-level association with diagnostic correctness but did not reliably identify the correct diagnosis as the top-ranked candidate within an individual list. The present findings therefore do not support conditional perplexity as a stand-alone reranking or post-generation filtering rule; rather, it may warrant further investigation as one component of broader output-level auditing or multimodal rescoring approaches.

The comparison across source models is also informative. GPT-4 had the highest numerical rate of including the correct diagnosis in the differential diagnosis list and showed the lowest median conditional perplexity score for correct diagnoses and the highest candidate-level AUC among the 3 models. One possible interpretation is that GPT-4–generated diagnosis strings were, on average, more closely aligned with the information captured in the case descriptions. Another possibility is that the diagnosis string used by GPT-4 was more naturally scored by the independent evaluator. Either way, the findings indicate that conditional perplexity score reflects properties of both narrative-to-diagnosis compatibility and language-model representation.

In paired analyses, the conditional perplexity scores for correct diagnoses generated by LLaMA2 and GPT-4 did not differ significantly from those of the case report–defined final diagnoses, whereas Bard showed significantly higher scores. This finding indicates similar conditional predictability under the scoring model for the former 2 source models but should not be interpreted as evidence of equivalent case-specific clinical compatibility.

Additionally, the discrimination achieved by conditional perplexity score was moderate rather than near perfect. The ROC curves showed substantial overlap between correct and incorrect diagnoses, and some incorrect diagnoses still received low scores. This behavior is expected in real-world differential diagnosis, where several incorrect differential diagnoses may nonetheless be partially compatible with the presenting syndrome [35]. Accordingly, the conditional perplexity score should be interpreted as a supportive output-level auditing signal rather than as a binary evaluation. In the context of XAI and uncertainty-aware decision support, such an externally computable score may add transparency without being mistaken for a complete explanation of clinical reasoning [25,36].

These results align with the broader literature on LLMs in medicine, which shows strong promise in benchmark-style tasks while also highlighting the need for better evaluation of grounding, interpretability, and uncertainty [48]. Recent work on hallucination and LLM reliability has distinguished data-driven or familiarity-based signals from reasoning-driven components [49], a distinction that is directly relevant here because conditional perplexity is expected to track model familiarity and lexical predictability as well as conditional predictability. More broadly, output-based evaluations across multiple LLMs have demonstrated that externally defined quantitative measures can identify systematic differences in model-generated outputs that are not captured by conventional performance benchmarks [50]. Although that work evaluated value-priority profiles rather than diagnostic reasoning, it illustrates the broader utility of output-level auditing across models. The present approach similarly applies an external quantitative measure to outputs from multiple source LLMs, but conditional perplexity remains task-specific and scorer-dependent. Similarly, calls for retrieval-grounded and verifiability-oriented evaluation of clinical LLM outputs emphasize that model-native scores should be interpreted alongside external evidence and subgroup or robustness audits rather than as stand-alone proof of validity [51].

### Limitations

Several limitations should be acknowledged. First, this was a retrospective study using published case reports from a single journal and a single publication year, which may overrepresent uncommon, educational, or diagnostically polished cases. Published case reports are written after the diagnosis is known and may contain lexical cues that foreshadow the final diagnosis. As a result, the findings may not generalize to routine clinical reasoning, unfiltered electronic health record data, or prospective settings in which diagnostic information is incomplete, noisy, abbreviated, or evolving [52].

Second, the analysis depended on diagnosis strings and prior physician verification in the previous dataset. Synonym handling, disease granularity, and adjudication choices may influence whether a generated diagnosis is counted as correct.

Third, the scorer model was Qwen2.5‐1.5B, a general-purpose LLM rather than a clinically specialized probability model, and conditional perplexity score may be influenced by diagnostic wording length, lexical frequency, or stylistic alignment in addition to clinical content [53]. Although post hoc analyses adjusted for diagnosis-string length, lexical overlap, exact phrase occurrence, and a candidate-corpus token-frequency proxy, these analyses could not measure the true token frequency in the scorer model’s pretraining distribution. Because only one independent scorer model was used, the robustness of the signal across different scorer architectures, larger models, and biomedical-domain scorers remains unknown.

Fourth, scorer-model contamination cannot be excluded. Qwen2.5‐1.5B may have been pretrained on some published case reports or related web content, and low perplexity for the correct diagnosis could partly reflect memorization or prior exposure rather than generalizable case-specific reasoning. Future studies should include contamination checks, held-out cases published after the scorer model training cutoff, or deidentified prospective clinical notes unavailable during pretraining.

Fifth, false-positive diagnoses in a differential diagnosis list are not necessarily nonsensical. Many may be partially compatible with alternatives that a clinician would reasonably entertain. In actual clinical practice, clinicians often intentionally include low-probability diagnoses when they represent life-threatening or require urgent exclusion [54]. This likely lowers the apparent discrimination of the metric.

Sixth, the study evaluated text compatibility rather than clinical utility, so the findings should not be directly interpreted as demonstrating improved patient outcomes or safe autonomous diagnosis [55,56]. Case-level cluster-bootstrap sensitivity analyses were added to address nesting by case, but candidate-level pooled statistics and multiple pairwise tests remain exploratory and hypothesis-generating.

Finally, the rapid evolution of LLMs should be considered when interpreting these findings. Because the models examined in this study have advanced substantially, including Bard’s transition to Gemini [57], the release of LLaMA4 [58], and the introduction of GPT-5 [59], the present results may not directly extend to newer generations of LLMs.

### Future Directions

Despite these limitations, the findings support the conditional perplexity score as a promising quantitative measure for benchmarking diagnostic compatibility in case-report datasets. It adds information beyond binary inclusion, aligns directionally with physician-verified diagnostic status, and can be applied uniformly across differential diagnosis lists from multiple LLMs. Future studies should build on the post hoc sensitivity analyses by using scorer-model token frequencies or unconditional diagnosis-string likelihoods, masking diagnosis-revealing terms, matching correct and incorrect strings on length and frequency, and scoring diagnoses against mismatched case descriptions as a negative control. Additional validation should examine medically specialized scorer models, ontology-normalized diagnosis strings, combined rescoring strategies, and unfiltered real-world clinical documentation such as electronic health record data, where narratives are abbreviated, incomplete, and less retrospectively polished [60,61]. It will also be important to determine whether this approach can be extended beyond free-text diagnostic strings to structured diagnostic representations, including International Classification of Diseases codes [62,63], the systematized nomenclature of medicine clinical terms [64], and, in Japan, Diagnosis Procedure Combination-related coding systems [65], to improve standardization and reduce ambiguity in diagnosis matching. Because diagnosis is not determined by a single score alone, future research should also investigate how conditional perplexity score can be integrated with CDSS benchmarks and established diagnostic evaluation frameworks, so that compatibility scoring is interpreted as one component of a broader diagnostic assessment rather than as a standalone indicator.

### Conclusions

Conditional perplexity score derived from an independent LLM provided a moderate signal separating case report–defined and physician-verified correct generated diagnoses from incorrect generated diagnoses in a large set of curated published case reports. The signal was consistent across 3 source LLMs and strongest for GPT-4–generated differential diagnoses, but the magnitude of discrimination was moderate. These findings support conditional perplexity score as a promising quantitative adjunct for ranking, auditing, and studying LLM-generated differential diagnoses, while highlighting the need for prospective validation in broader and less curated clinical datasets.

## Supplementary material

10.2196/98819 Multimedia Appendix 1 Sample Python code to calculate conditional perplexity scores.

10.2196/98819 Multimedia Appendix 2 Sample Python code for statistical analysis and post hoc sensitivity analyses.

10.2196/98819 Multimedia Appendix 3 Supplemental sensitivity analyses addressing lexical overlap, adjusted models, case-level cluster-bootstrap area under the receiver operating characteristic curves, and within-case comparator analyses.

## Abbreviations

AUC: area under the receiver operating characteristic curve
CDSS: clinical decision support system
CPU: central processing unit
GPU: graphics processing unit
LLM: large language model
NLL: negative log-likelihood
R-IDEA: revised-IDEA
ROC: receiver operating characteristic
ROC: receiver operating characteristic
XAI: explainable artificial intelligence
