# Supplementary material for: Conditional Perplexity Scoring for Large Language Model−Generated Differential Diagnoses in Case Reports: Preliminary Computational Evaluation
Source: JMIR Form Res. 2026 Sep 9;10:e98819. doi: 10.2196/98819 (PMC13557325; doi:10.2196/98819)
Supplement: Supplementary file 1 [file formative-v10-e98819-s001.docx]

Supplemental Table 1. Sample python code to calculate conditional perplexity scores

\# Computational Environment

\# Processor: 12th Gen Intel(R) Core(TM) i7-12700F (2.10 GHz)

\# Memory (RAM): 16 GB

\# Graphics Processing Unit: NVIDIA GeForce RTX 3060

\# Storage: 954 GB

import os

import math

import pandas as pd

import torch

import torch.nn.functional as F

from tqdm import tqdm

from transformers import AutoTokenizer, AutoModelForCausalLM

input_folder = PATH1

output_folder = PATH2

os.makedirs(output_folder, exist_ok=True)

model_name = "Qwen/Qwen2.5-1.5B"

device = torch.device("cuda" if torch.cuda.is_available() else "cpu")

if torch.cuda.is_available():

torch_dtype = torch.bfloat16 if torch.cuda.is_bf16_supported() else torch.float16

else:

torch_dtype = torch.float32

tokenizer = AutoTokenizer.from_pretrained(model_name, use_fast=True)

model = AutoModelForCausalLM.from_pretrained(

model_name,

torch_dtype=torch_dtype,

low_cpu_mem_usage=True,

)

model.to(device)

model.eval()

MAX_CONTEXT = getattr(model.config, "max_position_embeddings", 32768)

STRIDE = min(4096, MAX_CONTEXT // 2)

CASE_COL = "final_case"

GOLD_DX_COLS = [

"Final diagnosis1",

"Final diagnosis2",

"final diagnosis34", # kept exactly as in your real header

]

LLAMA_COLS = [f"DDx{i}_llama2" for i in range(1, 11)]

BARD_COLS = [f"DDx{i}_Bard" for i in range(1, 11)]

GPT_COLS	= [f"DDx{i}_GPT" for i in range(1, 11)]

def is_valid_text(x):

return isinstance(x, str) and len(x.strip()) > 0

def clean_text(x):

if pd.isna(x):

return None

if not isinstance(x, str):

x = str(x)

x = x.strip()

return x if x else None

def get_existing_columns(df, cols):

return [c for c in cols if c in df.columns]

def collect_gold_diagnoses(row, gold_cols):

diagnoses = []

seen = set()

for col in gold_cols:

if col in row.index:

val = clean_text(row[col])

if val is not None:

key = val.lower()

if key not in seen:

diagnoses.append(val)

seen.add(key)

return diagnoses

def combine_gold_for_joint_scoring(gold_diagnoses):

if not gold_diagnoses:

return None

return "; ".join(gold_diagnoses)

def build_prompt(clinical_text: str):

return f"""Clinical information from a case report:

{clinical_text}

The final diagnosis is """

def token_len(text: str):

if not is_valid_text(text):

return 0

return len(tokenizer(text, add_special_tokens=False)["input_ids"])

def conditional_nll_ppl_full_context(prompt: str, target: str):

"""

Exact conditional scoring in one forward pass when prompt+target fits in context.

Scores only the target tokens.

"""

prompt_ids = tokenizer(prompt, add_special_tokens=False, return_tensors="pt")["input_ids"]

target_ids = tokenizer(target, add_special_tokens=False, return_tensors="pt")["input_ids"]

full_ids = torch.cat([prompt_ids, target_ids], dim=1).to(device)

prompt_len = prompt_ids.size(1)

target_len = target_ids.size(1)

labels = full_ids.clone()

labels[:, :prompt_len] = -100

with torch.no_grad():

outputs = model(full_ids, labels=labels)

avg_nll = outputs.loss.item()

ppl = math.exp(avg_nll)

total_logprob = -avg_nll * target_len

return {

"prompt_tokens": int(prompt_len),

"target_tokens": int(target_len),

"total_tokens": int(full_ids.size(1)),

"avg_nll": float(avg_nll),

"ppl": float(ppl),

"total_logprob": float(total_logprob),

"mode": "full_context",

"fits_in_context": True,

}

def conditional_nll_ppl_strided(prompt: str, target: str, stride: int = STRIDE):

"""

Approximate conditional scoring for sequences longer than model context.

Scores only target tokens, using a sliding window.

"""

prompt_ids = tokenizer(prompt, add_special_tokens=False, return_tensors="pt")["input_ids"]

target_ids = tokenizer(target, add_special_tokens=False, return_tensors="pt")["input_ids"]

full_ids = torch.cat([prompt_ids, target_ids], dim=1).to(device)

prompt_len = prompt_ids.size(1)

target_len = target_ids.size(1)

seq_len = full_ids.size(1)

if target_len == 0:

return None

nll_sum = 0.0

n_target_tokens = 0

prev_end_loc = 0

for begin_loc in range(0, seq_len, stride):

end_loc = min(begin_loc + MAX_CONTEXT, seq_len)

input_ids = full_ids[:, begin_loc:end_loc]

with torch.no_grad():

logits = model(input_ids).logits

\# Logits at local position j predict token j+1.

\# Only score target tokens, and do not double-count overlaps.

score_start_global = max(begin_loc + 1, prompt_len, prev_end_loc)

score_end_global = end_loc

if score_start_global < score_end_global:

local_label_start = score_start_global - begin_loc

local_label_end = score_end_global - begin_loc

shift_logits = logits[:, local_label_start - 1:local_label_end - 1, :]

shift_labels = input_ids[:, local_label_start:local_label_end]

log_probs = F.log_softmax(shift_logits, dim=-1)

token_log_probs = log_probs.gather(-1, shift_labels.unsqueeze(-1)).squeeze(-1)

nll_sum += (-token_log_probs).sum().item()

n_target_tokens += shift_labels.numel()

prev_end_loc = end_loc

if end_loc == seq_len:

break

if n_target_tokens == 0:

return None

avg_nll = nll_sum / n_target_tokens

ppl = math.exp(avg_nll)

total_logprob = -nll_sum

return {

"prompt_tokens": int(prompt_len),

"target_tokens": int(target_len),

"total_tokens": int(seq_len),

"avg_nll": float(avg_nll),

"ppl": float(ppl),

"total_logprob": float(total_logprob),

"mode": "strided",

"fits_in_context": False,

}

def conditional_nll_ppl(prompt: str, target: str):

if not is_valid_text(prompt) or not is_valid_text(target):

return None

prompt_n = token_len(prompt)

target_n = token_len(target)

total_n = prompt_n + target_n

if total_n <= MAX_CONTEXT:

return conditional_nll_ppl_full_context(prompt, target)

else:

return conditional_nll_ppl_strided(prompt, target)

def score_candidates(clinical_text, candidates):

prompt = build_prompt(clinical_text)

results = []

for cand in candidates:

cand = clean_text(cand)

if cand is None:

continue

score = conditional_nll_ppl(prompt, cand)

if score is None:

continue

results.append({

"candidate": cand,

"target_tokens": score["target_tokens"],

"avg_nll": score["avg_nll"],

"ppl": score["ppl"],

"total_logprob": score["total_logprob"],

"mode": score["mode"],

})

results.sort(key=lambda x: x["avg_nll"])

return results

def find_best_exact_gold_rank(ranked_candidates, gold_diagnoses):

gold_set = {g.strip().lower() for g in gold_diagnoses if is_valid_text(g)}

for i, item in enumerate(ranked_candidates, start=1):

cand = item["candidate"].strip().lower()

if cand in gold_set:

return i

return None

for filename in os.listdir(input_folder):

if not filename.endswith(".csv"):

continue

input_path = os.path.join(input_folder, filename)

print(f"Processing {filename}...")

df = pd.read_csv(input_path)

if CASE_COL not in df.columns:

print(f"Skipping {filename}: missing clinical text column '{CASE_COL}'")

continue

llama_cols_existing = get_existing_columns(df, LLAMA_COLS)

bard_cols_existing = get_existing_columns(df, BARD_COLS)

gpt_cols_existing = get_existing_columns(df, GPT_COLS)

\# Output columns

final_case_tokens_col = []

gold_list_col = []

gold_combined_col = []

gold_joint_prompt_tokens = []

gold_joint_target_tokens = []

gold_joint_total_tokens = []

gold_joint_scoring_mode = []

gold_joint_avg_nll = []

gold_joint_ppl = []

gold_joint_total_logprob = []

gold1_avg_nll = []

gold1_ppl = []

gold2_avg_nll = []

gold2_ppl = []

gold3_avg_nll = []

gold3_ppl = []

llama_best_candidate = []

llama_best_avg_nll = []

llama_best_ppl = []

llama_best_mode = []

llama_gold_rank = []

bard_best_candidate = []

bard_best_avg_nll = []

bard_best_ppl = []

bard_best_mode = []

bard_gold_rank = []

gpt_best_candidate = []

gpt_best_avg_nll = []

gpt_best_ppl = []

gpt_best_mode = []

gpt_gold_rank = []

for _, row in tqdm(df.iterrows(), total=len(df), desc=f"→ {filename}"):

clinical_text = clean_text(row[CASE_COL])

if clinical_text is None:

final_case_tokens_col.append(None)

gold_list_col.append(None)

gold_combined_col.append(None)

gold_joint_prompt_tokens.append(None)

gold_joint_target_tokens.append(None)

gold_joint_total_tokens.append(None)

gold_joint_scoring_mode.append(None)

gold_joint_avg_nll.append(None)

gold_joint_ppl.append(None)

gold_joint_total_logprob.append(None)

gold1_avg_nll.append(None)

gold1_ppl.append(None)

gold2_avg_nll.append(None)

gold2_ppl.append(None)

gold3_avg_nll.append(None)

gold3_ppl.append(None)

llama_best_candidate.append(None)

llama_best_avg_nll.append(None)

llama_best_ppl.append(None)

llama_best_mode.append(None)

llama_gold_rank.append(None)

bard_best_candidate.append(None)

bard_best_avg_nll.append(None)

bard_best_ppl.append(None)

bard_best_mode.append(None)

bard_gold_rank.append(None)

gpt_best_candidate.append(None)

gpt_best_avg_nll.append(None)

gpt_best_ppl.append(None)

gpt_best_mode.append(None)

gpt_gold_rank.append(None)

continue

final_case_tokens_col.append(token_len(clinical_text))

gold_diagnoses = collect_gold_diagnoses(row, GOLD_DX_COLS)

gold_combined = combine_gold_for_joint_scoring(gold_diagnoses)

gold_list_col.append(" | ".join(gold_diagnoses) if gold_diagnoses else None)

gold_combined_col.append(gold_combined)

prompt = build_prompt(clinical_text)

if gold_combined is not None:

s_joint = conditional_nll_ppl(prompt, gold_combined)

else:

s_joint = None

if s_joint is None:

gold_joint_prompt_tokens.append(None)

gold_joint_target_tokens.append(None)

gold_joint_total_tokens.append(None)

gold_joint_scoring_mode.append(None)

gold_joint_avg_nll.append(None)

gold_joint_ppl.append(None)

gold_joint_total_logprob.append(None)

else:

gold_joint_prompt_tokens.append(s_joint["prompt_tokens"])

gold_joint_target_tokens.append(s_joint["target_tokens"])

gold_joint_total_tokens.append(s_joint["total_tokens"])

gold_joint_scoring_mode.append(s_joint["mode"])

gold_joint_avg_nll.append(s_joint["avg_nll"])

gold_joint_ppl.append(s_joint["ppl"])

gold_joint_total_logprob.append(s_joint["total_logprob"])

individual_gold_vals = []

for c in GOLD_DX_COLS:

individual_gold_vals.append(clean_text(row[c]) if c in row.index else None)

individual_gold_scores = []

for dx in individual_gold_vals:

if dx is None:

individual_gold_scores.append(None)

else:

individual_gold_scores.append(conditional_nll_ppl(prompt, dx))

gold1_avg_nll.append(None if individual_gold_scores[0] is None else individual_gold_scores[0]["avg_nll"])

gold1_ppl.append(None if individual_gold_scores[0] is None else individual_gold_scores[0]["ppl"])

gold2_avg_nll.append(None if individual_gold_scores[1] is None else individual_gold_scores[1]["avg_nll"])

gold2_ppl.append(None if individual_gold_scores[1] is None else individual_gold_scores[1]["ppl"])

gold3_avg_nll.append(None if individual_gold_scores[2] is None else individual_gold_scores[2]["avg_nll"])

gold3_ppl.append(None if individual_gold_scores[2] is None else individual_gold_scores[2]["ppl"])

llama_candidates = [row[c] for c in llama_cols_existing]

llama_ranked = score_candidates(clinical_text, llama_candidates)

if len(llama_ranked) == 0:

llama_best_candidate.append(None)

llama_best_avg_nll.append(None)

llama_best_ppl.append(None)

llama_best_mode.append(None)

llama_gold_rank.append(None)

else:

llama_best_candidate.append(llama_ranked[0]["candidate"])

llama_best_avg_nll.append(llama_ranked[0]["avg_nll"])

llama_best_ppl.append(llama_ranked[0]["ppl"])

llama_best_mode.append(llama_ranked[0]["mode"])

llama_gold_rank.append(find_best_exact_gold_rank(llama_ranked, gold_diagnoses))

bard_candidates = [row[c] for c in bard_cols_existing]

bard_ranked = score_candidates(clinical_text, bard_candidates)

if len(bard_ranked) == 0:

bard_best_candidate.append(None)

bard_best_avg_nll.append(None)

bard_best_ppl.append(None)

bard_best_mode.append(None)

bard_gold_rank.append(None)

else:

bard_best_candidate.append(bard_ranked[0]["candidate"])

bard_best_avg_nll.append(bard_ranked[0]["avg_nll"])

bard_best_ppl.append(bard_ranked[0]["ppl"])

bard_best_mode.append(bard_ranked[0]["mode"])

bard_gold_rank.append(find_best_exact_gold_rank(bard_ranked, gold_diagnoses))

gpt_candidates = [row[c] for c in gpt_cols_existing]

gpt_ranked = score_candidates(clinical_text, gpt_candidates)

if len(gpt_ranked) == 0:

gpt_best_candidate.append(None)

gpt_best_avg_nll.append(None)

gpt_best_ppl.append(None)

gpt_best_mode.append(None)

gpt_gold_rank.append(None)

else:

gpt_best_candidate.append(gpt_ranked[0]["candidate"])

gpt_best_avg_nll.append(gpt_ranked[0]["avg_nll"])

gpt_best_ppl.append(gpt_ranked[0]["ppl"])

gpt_best_mode.append(gpt_ranked[0]["mode"])

gpt_gold_rank.append(find_best_exact_gold_rank(gpt_ranked, gold_diagnoses))

df["final_case_tokens"] = final_case_tokens_col

df["gold_diagnoses_list"] = gold_list_col

df["gold_diagnoses_combined"] = gold_combined_col

df["gold_joint_prompt_tokens"] = gold_joint_prompt_tokens

df["gold_joint_target_tokens"] = gold_joint_target_tokens

df["gold_joint_total_tokens"] = gold_joint_total_tokens

df["gold_joint_scoring_mode"] = gold_joint_scoring_mode

df["gold_joint_avg_nll"] = gold_joint_avg_nll

df["gold_joint_conditional_ppl"] = gold_joint_ppl

df["gold_joint_total_logprob"] = gold_joint_total_logprob

df["gold1_avg_nll"] = gold1_avg_nll

df["gold1_conditional_ppl"] = gold1_ppl

df["gold2_avg_nll"] = gold2_avg_nll

df["gold2_conditional_ppl"] = gold2_ppl

df["gold3_avg_nll"] = gold3_avg_nll

df["gold3_conditional_ppl"] = gold3_ppl

df["llama_best_candidate_by_avg_nll"] = llama_best_candidate

df["llama_best_candidate_avg_nll"] = llama_best_avg_nll

df["llama_best_candidate_ppl"] = llama_best_ppl

df["llama_best_candidate_mode"] = llama_best_mode

df["llama_gold_exact_rank"] = llama_gold_rank

df["bard_best_candidate_by_avg_nll"] = bard_best_candidate

df["bard_best_candidate_avg_nll"] = bard_best_avg_nll

df["bard_best_candidate_ppl"] = bard_best_ppl

df["bard_best_candidate_mode"] = bard_best_mode

df["bard_gold_exact_rank"] = bard_gold_rank

df["gpt_best_candidate_by_avg_nll"] = gpt_best_candidate

df["gpt_best_candidate_avg_nll"] = gpt_best_avg_nll

df["gpt_best_candidate_ppl"] = gpt_best_ppl

df["gpt_best_candidate_mode"] = gpt_best_mode

df["gpt_gold_exact_rank"] = gpt_gold_rank

output_path = os.path.join(output_folder, filename)

df.to_csv(output_path, index=False)

print(f"Saved: {output_path}")
