# Supplementary material for: Conditional Perplexity Scoring for Large Language Model−Generated Differential Diagnoses in Case Reports: Preliminary Computational Evaluation
Source: JMIR Form Res. 2026 Sep 9;10:e98819. doi: 10.2196/98819 (PMC13557325; doi:10.2196/98819)
Supplement: Supplementary file 2 [file formative-v10-e98819-s002.docx]

Supplemental Table 2. Sample Python code for statistical analysis

from pathlib import Path

from typing import Dict, Iterable, List, Tuple

import matplotlib.pyplot as plt

import numpy as np

import pandas as pd

from scipy.stats import kruskal, mannwhitneyu, wilcoxon

from sklearn.metrics import auc, roc_auc_score, roc_curve

INPUT_CSV = Path3

OUTDIR = Path4

OUTDIR.mkdir(parents=True, exist_ok=True)

pd.set_option('display.max_columns', 200)

pd.set_option('display.width', 200)

MODEL_CONFIG = {

'llama2': {

'display': 'LLaMA2',

'inclusion_col': 'inclusion_llama2',

'rank_col': 'rank_llama2',

'correct_ppl_col': 'llama2_Correct_DDx_PPL',

'avg_incorrect_case_col': 'llama2_Avg_Incorrect_DDx_PPL',

},

'Bard': {

'display': 'Bard',

'inclusion_col': 'inclusion_Bard',

'rank_col': 'rank_Bard',

'correct_ppl_col': 'Bard_Correct_DDx_PPL',

'avg_incorrect_case_col': 'Bard_Avg_Incorrect_DDx_PPL',

},

'GPT': {

'display': 'GPT',

'inclusion_col': 'inclusion_GPT',

'rank_col': 'rank_GPT',

'correct_ppl_col': 'GPT_Correct_DDx_PPL',

'avg_incorrect_case_col': 'GPT_Avg_Incorrect_DDx_PPL',

},

}

REQUIRED_COLUMNS = [

'Final_diagnosis1_PPL',

'Final diagnosis1',

'Final diagnosis2',

'final diagnosis34',

]

for model in MODEL_CONFIG:

REQUIRED_COLUMNS.extend([

MODEL_CONFIG[model]['inclusion_col'],

MODEL_CONFIG[model]['rank_col'],

MODEL_CONFIG[model]['correct_ppl_col'],

MODEL_CONFIG[model]['avg_incorrect_case_col'],

])

for i in range(1, 11):

REQUIRED_COLUMNS.extend([

f'DDx{i}_{model}',

f'DDx{i}_{model}_PPL',

f'DDx{i}_{model}_avg_NLL',

])

def ensure_required_columns(df: pd.DataFrame) -> None:

missing = [col for col in REQUIRED_COLUMNS if col not in df.columns]

if missing:

raise ValueError(f'Missing required columns: {missing}')

def is_nonempty(value: object) -> bool:

if pd.isna(value):

return False

return str(value).strip() != ''

def clean_text(value: object) -> str:

if pd.isna(value):

return ''

return str(value).strip().lower()

def median_iqr(values: Iterable[float]) -> Dict[str, float]:

arr = np.asarray(list(values), dtype=float)

if arr.size == 0:

return {'n': 0, 'median': np.nan, 'q1': np.nan, 'q3': np.nan}

q1, q3 = np.quantile(arr, [0.25, 0.75])

return {

'n': int(arr.size),

'median': float(np.median(arr)),

'q1': float(q1),

'q3': float(q3),

}

def read_csv_robust(path: Path) -> tuple[pd.DataFrame, str]:

encodings_to_try = [

'utf-8',

'utf-8-sig',

'cp932',

'shift_jis',

'cp1252',

'latin1',

'utf-16',

]

last_error = None

for encoding in encodings_to_try:

try:

df = pd.read_csv(path, encoding=encoding)

return df, encoding

except UnicodeDecodeError as e:

last_error = e

except Exception:

pass

df = pd.read_csv(path, encoding='utf-8', encoding_errors='replace')

return df, 'utf-8 (encoding_errors="replace")'

def bootstrap_auc_ci(y_true, scores, n_boot=2000, alpha=0.95, seed=42):

"""

Bootstrap 95% CI for AUROC.

y_true: 1 = positive class, 0 = negative class

scores: higher score = more likely positive class

For PPL, use -PPL because lower PPL is better.

"""

from sklearn.metrics import roc_auc_score

y_true = np.asarray(y_true)

scores = np.asarray(scores)

mask = np.isfinite(y_true) & np.isfinite(scores)

y_true = y_true[mask]

scores = scores[mask]

auc_obs = roc_auc_score(y_true, scores)

rng = np.random.default_rng(seed)

boot_aucs = []

n = len(y_true)

for _ in range(n_boot):

idx = rng.integers(0, n, n)

y_b = y_true[idx]

s_b = scores[idx]

\# Need both classes present

if len(np.unique(y_b)) < 2:

continue

boot_aucs.append(roc_auc_score(y_b, s_b))

lower_q = (1 - alpha) / 2

upper_q = 1 - lower_q

ci_low, ci_high = np.quantile(boot_aucs, [lower_q, upper_q])

return {

"auc": float(auc_obs),

"ci_low": float(ci_low),

"ci_high": float(ci_high),

"n_boot_valid": int(len(boot_aucs)),

}

def bootstrap_median_ci(values, n_boot=2000, alpha=0.95, seed=42):

arr = pd.Series(values).dropna().astype(float).to_numpy()

if len(arr) == 0:

return {"median": np.nan, "ci_low": np.nan, "ci_high": np.nan, "n": 0}

median_obs = float(np.median(arr))

rng = np.random.default_rng(seed)

boot_medians = []

n = len(arr)

for _ in range(n_boot):

sample = arr[rng.integers(0, n, n)]

boot_medians.append(np.median(sample))

lower_q = (1 - alpha) / 2

upper_q = 1 - lower_q

ci_low, ci_high = np.quantile(boot_medians, [lower_q, upper_q])

return {

"median": median_obs,

"ci_low": float(ci_low),

"ci_high": float(ci_high),

"n": int(n),

}

def build_candidate_level_dataframe(df: pd.DataFrame) -> pd.DataFrame:

rows: List[Dict[str, object]] = []

for case_idx, row in df.iterrows():

for model, cfg in MODEL_CONFIG.items():

inclusion = int(row[cfg['inclusion_col']]) if pd.notna(row[cfg['inclusion_col']]) else 0

rank = int(row[cfg['rank_col']]) if pd.notna(row[cfg['rank_col']]) else 0

for pos in range(1, 11):

diag_col = f'DDx{pos}_{model}'

ppl_col = f'DDx{pos}_{model}_PPL'

nll_col = f'DDx{pos}_{model}_avg_NLL'

diagnosis = row[diag_col]

ppl = row[ppl_col]

avg_nll = row[nll_col]

if not is_nonempty(diagnosis) or pd.isna(ppl):

continue

is_correct = bool(inclusion == 1 and rank == pos)

rows.append({

'case_id': case_idx,

'model': model,

'model_display': cfg['display'],

'candidate_position': pos,

'diagnosis': str(diagnosis).strip(),

'ppl': float(ppl),

'avg_nll': float(avg_nll) if pd.notna(avg_nll) else np.nan,

'is_correct_generated': is_correct,

'case_has_correct_generated': bool(inclusion == 1),

'original_rank_of_correct': rank if inclusion == 1 else np.nan,

})

out = pd.DataFrame(rows)

if out.empty:

raise ValueError('No candidate-level rows were constructed from the CSV.')

return out

def build_gold_standard_dataframe(df: pd.DataFrame) -> pd.DataFrame:

gold_rows: List[Dict[str, object]] = []

for case_idx, row in df.iterrows():

ppl = row['Final_diagnosis1_PPL']

diagnosis = row['Final diagnosis1']

if is_nonempty(diagnosis) and pd.notna(ppl):

gold_rows.append({

'case_id': case_idx,

'diagnosis': str(diagnosis).strip(),

'ppl': float(ppl),

})

return pd.DataFrame(gold_rows)

def coverage_table(df: pd.DataFrame) -> pd.DataFrame:

rows = []

n_cases = len(df)

for model, cfg in MODEL_CONFIG.items():

inclusion = df[cfg['inclusion_col']].fillna(0).astype(int)

rank = df[cfg['rank_col']].fillna(0).astype(int)

rows.append({

'model': cfg['display'],

'cases_with_correct_ddx': int((inclusion == 1).sum()),

'coverage_pct': float((inclusion == 1).mean() * 100),

'original_top1_cases': int(((inclusion == 1) & (rank == 1)).sum()),

'original_top1_pct': float((((inclusion == 1) & (rank == 1)).mean()) * 100),

'original_top3_cases': int(((inclusion == 1) & (rank <= 3) & (rank >= 1)).sum()),

'original_top3_pct': float((((inclusion == 1) & (rank <= 3) & (rank >= 1)).mean()) * 100),

'total_cases': n_cases,

})

return pd.DataFrame(rows)

def summary_table(gold_df: pd.DataFrame, candidates: pd.DataFrame) -> pd.DataFrame:

gold_stats = median_iqr(gold_df['ppl'])

correct_stats = median_iqr(candidates.loc[candidates['is_correct_generated'], 'ppl'])

incorrect_stats = median_iqr(candidates.loc[~candidates['is_correct_generated'], 'ppl'])

rows = []

for label, stats in [

('Gold standard final diagnoses', gold_stats),

('Correct generated diagnoses', correct_stats),

('Incorrect generated diagnoses', incorrect_stats),

]:

rows.append({

'group': label,

'n': stats['n'],

'median_ppl': stats['median'],

'q1': stats['q1'],

'q3': stats['q3'],

})

return pd.DataFrame(rows)

def mann_whitney_summary(a: np.ndarray, b: np.ndarray, label_a: str, label_b: str) -> Dict[str, object]:

result = mannwhitneyu(a, b, alternative='two-sided')

return {

'comparison': f'{label_a} vs {label_b}',

'u_statistic': float(result.statistic),

'p_value': float(result.pvalue),

}

def pooled_and_model_specific_tests(gold_df: pd.DataFrame, candidates: pd.DataFrame):

test_rows = []

gold = gold_df['ppl'].to_numpy(dtype=float)

correct_all = candidates.loc[candidates['is_correct_generated'], 'ppl'].to_numpy(dtype=float)

incorrect_all = candidates.loc[~candidates['is_correct_generated'], 'ppl'].to_numpy(dtype=float)

test_rows.append(mann_whitney_summary(gold, incorrect_all, 'Gold', 'Incorrect'))

test_rows.append(mann_whitney_summary(correct_all, incorrect_all, 'Correct generated', 'Incorrect'))

test_rows.append(mann_whitney_summary(gold, correct_all, 'Gold', 'Correct generated'))

model_rows = []

for model, cfg in MODEL_CONFIG.items():

mdf = candidates.loc[candidates['model'] == model].copy()

correct = mdf.loc[mdf['is_correct_generated'], 'ppl'].to_numpy(dtype=float)

incorrect = mdf.loc[~mdf['is_correct_generated'], 'ppl'].to_numpy(dtype=float)

mw = mannwhitneyu(correct, incorrect, alternative='two-sided')

model_rows.append({

'model': cfg['display'],

**{f'correct_{k}': v for k, v in median_iqr(correct).items()},

**{f'incorrect_{k}': v for k, v in median_iqr(incorrect).items()},

'mann_whitney_u': float(mw.statistic),

'mann_whitney_p': float(mw.pvalue),

})

kruskal_result = kruskal(*[

candidates.loc[

(candidates['model'] == model) & (candidates['is_correct_generated']), 'ppl'

].to_numpy(dtype=float)

for model in MODEL_CONFIG

])

test_rows.append({

'comparison': 'Correct generated diagnoses across models (Kruskal-Wallis)',

'u_statistic': float(kruskal_result.statistic),

'p_value': float(kruskal_result.pvalue),

})

return pd.DataFrame(test_rows), pd.DataFrame(model_rows)

def compute_auc_table(gold_df: pd.DataFrame, candidates: pd.DataFrame) -> pd.DataFrame:

rows = []

gold = gold_df['ppl'].to_numpy(dtype=float)

for model, cfg in MODEL_CONFIG.items():

mdf = candidates.loc[candidates['model'] == model].copy()

correct = mdf.loc[mdf['is_correct_generated'], 'ppl'].to_numpy(dtype=float)

incorrect = mdf.loc[~mdf['is_correct_generated'], 'ppl'].to_numpy(dtype=float)

same_case_incorrect = mdf.loc[

(~mdf['is_correct_generated']) & (mdf['case_has_correct_generated']), 'ppl'

].to_numpy(dtype=float)

rows.append({

'model': cfg['display'],

'auc_gold_vs_incorrect_full': float(

roc_auc_score(

np.r_[np.ones(len(gold)), np.zeros(len(incorrect))],

-np.r_[gold, incorrect],

)

),

'auc_correct_vs_incorrect_full': float(

roc_auc_score(

np.r_[np.ones(len(correct)), np.zeros(len(incorrect))],

-np.r_[correct, incorrect],

)

),

'auc_correct_vs_incorrect_same_case_subset': float(

roc_auc_score(

np.r_[np.ones(len(correct)), np.zeros(len(same_case_incorrect))],

-np.r_[correct, same_case_incorrect],

)

),

'n_correct': int(len(correct)),

'n_incorrect_full': int(len(incorrect)),

'n_incorrect_same_case_subset': int(len(same_case_incorrect)),

})

pooled_correct = candidates.loc[candidates['is_correct_generated'], 'ppl'].to_numpy(dtype=float)

pooled_incorrect = candidates.loc[~candidates['is_correct_generated'], 'ppl'].to_numpy(dtype=float)

rows.append({

'model': 'Pooled',

'auc_gold_vs_incorrect_full': float(

roc_auc_score(

np.r_[np.ones(len(gold)), np.zeros(len(pooled_incorrect))],

-np.r_[gold, pooled_incorrect],

)

),

'auc_correct_vs_incorrect_full': float(

roc_auc_score(

np.r_[np.ones(len(pooled_correct)), np.zeros(len(pooled_incorrect))],

-np.r_[pooled_correct, pooled_incorrect],

)

),

'auc_correct_vs_incorrect_same_case_subset': np.nan,

'n_correct': int(len(pooled_correct)),

'n_incorrect_full': int(len(pooled_incorrect)),

'n_incorrect_same_case_subset': np.nan,

})

return pd.DataFrame(rows)

def paired_case_table(df: pd.DataFrame) -> pd.DataFrame:

rows = []

gold = df['Final_diagnosis1_PPL'].astype(float)

for model, cfg in MODEL_CONFIG.items():

inclusion = df[cfg['inclusion_col']].fillna(0).astype(int)

mask = (

(inclusion == 1)

& df[cfg['correct_ppl_col']].notna()

& df[cfg['avg_incorrect_case_col']].notna()

& gold.notna()

)

correct = df.loc[mask, cfg['correct_ppl_col']].astype(float).to_numpy()

avg_incorrect = df.loc[mask, cfg['avg_incorrect_case_col']].astype(float).to_numpy()

gold_same_cases = gold.loc[mask].astype(float).to_numpy()

lower_count = int((correct < avg_incorrect).sum())

w1 = wilcoxon(correct, avg_incorrect, alternative='two-sided')

w2 = wilcoxon(gold_same_cases, correct, alternative='two-sided')

rows.append({

'model': cfg['display'],

'n_paired_cases': int(mask.sum()),

'correct_lt_avg_incorrect_n': lower_count,

'correct_lt_avg_incorrect_pct': float(lower_count / max(int(mask.sum()), 1) * 100),

'median_correct': float(np.median(correct)),

'median_avg_incorrect': float(np.median(avg_incorrect)),

'wilcoxon_correct_vs_avg_incorrect_stat': float(w1.statistic),

'wilcoxon_correct_vs_avg_incorrect_p': float(w1.pvalue),

'median_gold_same_cases': float(np.median(gold_same_cases)),

'wilcoxon_gold_vs_correct_stat': float(w2.statistic),

'wilcoxon_gold_vs_correct_p': float(w2.pvalue),

})

return pd.DataFrame(rows)

def save_roc_plot_gold_vs_incorrect(gold_df: pd.DataFrame, candidates: pd.DataFrame, outpath: Path) -> None:

gold = gold_df['ppl'].to_numpy(dtype=float)

plt.figure(figsize=(10, 8))

for model, cfg in MODEL_CONFIG.items():

incorrect = candidates.loc[

(candidates['model'] == model) & (~candidates['is_correct_generated']), 'ppl'

].to_numpy(dtype=float)

y_true = np.r_[np.ones(len(gold)), np.zeros(len(incorrect))]

scores = -np.r_[gold, incorrect]

fpr, tpr, _ = roc_curve(y_true, scores)

roc_auc = auc(fpr, tpr)

plt.plot(fpr, tpr, linewidth=2, label=f"{cfg['display']} Incorrects (AUC = {roc_auc:.3f})")

plt.plot([0, 1], [0, 1], '--', linewidth=2, label='Random Chance (AUC = 0.500)')

plt.xlabel('False Positive Rate (Incorrect Differentials)')

plt.ylabel('True Positive Rate (Gold Standard Diagnoses)')

plt.title('ROC Curve: Conditional PPL Distinguishing Diagnoses')

plt.legend(loc='lower right')

plt.grid(True, alpha=0.3)

plt.tight_layout()

plt.savefig(outpath, dpi=300)

plt.show()

plt.close()

def save_roc_plot_correct_vs_incorrect(candidates: pd.DataFrame, outpath: Path) -> None:

plt.figure(figsize=(10, 8))

for model, cfg in MODEL_CONFIG.items():

mdf = candidates.loc[candidates['model'] == model]

correct = mdf.loc[mdf['is_correct_generated'], 'ppl'].to_numpy(dtype=float)

incorrect = mdf.loc[~mdf['is_correct_generated'], 'ppl'].to_numpy(dtype=float)

y_true = np.r_[np.ones(len(correct)), np.zeros(len(incorrect))]

scores = -np.r_[correct, incorrect]

fpr, tpr, _ = roc_curve(y_true, scores)

roc_auc = auc(fpr, tpr)

plt.plot(fpr, tpr, linewidth=2, label=f"{cfg['display']} correct vs incorrect (AUC = {roc_auc:.3f})")

plt.plot([0, 1], [0, 1], '--', linewidth=2, label='Random Chance (AUC = 0.500)')

plt.xlabel('False Positive Rate (Incorrect Differentials)')

plt.ylabel('True Positive Rate (Correct Generated Diagnoses)')

plt.title('ROC Curve: Correct Generated vs Incorrect Diagnoses')

plt.legend(loc='lower right')

plt.grid(True, alpha=0.3)

plt.tight_layout()

plt.savefig(outpath, dpi=300)

plt.show()

plt.close()

def save_distribution_plot(gold_df: pd.DataFrame, candidates: pd.DataFrame, outpath: Path) -> None:

gold = gold_df['ppl'].to_numpy(dtype=float)

correct = candidates.loc[candidates['is_correct_generated'], 'ppl'].to_numpy(dtype=float)

incorrect = candidates.loc[~candidates['is_correct_generated'], 'ppl'].to_numpy(dtype=float)

groups = ['Gold final dx', 'Correct generated', 'Incorrect generated']

data = [gold, correct, incorrect]

plt.figure(figsize=(9, 7))

plt.boxplot(data, labels=groups, showfliers=False)

plt.yscale('log')

plt.ylabel('Conditional perplexity scores (log scale)')

plt.title('Conditional perplexity scores by diagnosis category')

plt.tight_layout()

plt.savefig(outpath, dpi=300)

plt.show()

plt.close()

gold_df = build_gold_standard_dataframe(df)

candidates = build_candidate_level_dataframe(df)

print('Gold-standard diagnosis rows:', len(gold_df))

print('Candidate-level rows:', len(candidates))

coverage = coverage_table(df)

summary = summary_table(gold_df, candidates)

tests, model_specific = pooled_and_model_specific_tests(gold_df, candidates)

auc_table = compute_auc_table(gold_df, candidates)

paired = paired_case_table(df)

coverage

median_rows = []

gold_ci = bootstrap_median_ci(df["Final_diagnosis1_PPL"])

median_rows.append({

"group": "Gold standard final diagnosis",

"n": gold_ci["n"],

"median": gold_ci["median"],

"ci_low": gold_ci["ci_low"],

"ci_high": gold_ci["ci_high"],

})

for model_key, cfg in MODEL_CONFIG.items():

model_name = cfg["display"]

corr_ci = bootstrap_median_ci(df[cfg["correct_ppl_col"]])

inc_ci = bootstrap_median_ci(df[cfg["avg_incorrect_case_col"]])

median_rows.append({

"group": f"{model_name} correct DDx",

"n": corr_ci["n"],

"median": corr_ci["median"],

"ci_low": corr_ci["ci_low"],

"ci_high": corr_ci["ci_high"],

})

median_rows.append({

"group": f"{model_name} avg incorrect DDx",

"n": inc_ci["n"],

"median": inc_ci["median"],

"ci_low": inc_ci["ci_low"],

"ci_high": inc_ci["ci_high"],

})

median_ci_df = pd.DataFrame(median_rows)

median_ci_df

summary

model_specific

tests

paired

auc_table

from sklearn.metrics import roc_auc_score

MODEL_SPECS = [

{

"model": "LLaMA2",

"rank_col": "rank_llama2",

"ppl_cols": [f"DDx{i}_llama2_PPL" for i in range(1, 11)],

},

{

"model": "Bard",

"rank_col": "rank_Bard",

"ppl_cols": [f"DDx{i}_Bard_PPL" for i in range(1, 11)],

},

{

"model": "GPT",

"rank_col": "rank_GPT",

"ppl_cols": [f"DDx{i}_GPT_PPL" for i in range(1, 11)],

},

]

df["Final_diagnosis1_PPL"] = pd.to_numeric(df["Final_diagnosis1_PPL"], errors="coerce")

for spec in MODEL_SPECS:

df[spec["rank_col"]] = pd.to_numeric(df[spec["rank_col"]], errors="coerce")

for c in spec["ppl_cols"]:

df[c] = pd.to_numeric(df[c], errors="coerce")

def collect_model_candidate_level_lists(df, rank_col, ppl_cols):

correct_list = []

incorrect_full_list = []

incorrect_same_case_list = []

for idx in df.index:

rank_val = df.at[idx, rank_col]

ppl_vals = [df.at[idx, c] for c in ppl_cols]

valid_pairs = [

(pos, val) for pos, val in enumerate(ppl_vals, start=1)

if pd.notna(val)

]

has_correct = pd.notna(rank_val) and int(rank_val) in range(1, 11)

correct_pos = int(rank_val) if has_correct else None

if has_correct:

correct_val = ppl_vals[correct_pos - 1]

if pd.notna(correct_val):

correct_list.append(float(correct_val))

for pos, val in valid_pairs:

is_correct_candidate = has_correct and (pos == correct_pos)

\# Full incorrect set: all individual incorrect candidates

if not is_correct_candidate:

incorrect_full_list.append(float(val))

\# Same-case subset: only incorrect candidates from rows with a correct DDx

if has_correct and (not is_correct_candidate):

incorrect_same_case_list.append(float(val))

return correct_list, incorrect_full_list, incorrect_same_case_list

auc_rows = []

for spec in MODEL_SPECS:

model_name = spec["model"]

rank_col = spec["rank_col"]

ppl_cols = spec["ppl_cols"]

correct_list, incorrect_full_list, incorrect_same_case_list = collect_model_candidate_level_lists(

df=df,

rank_col=rank_col,

ppl_cols=ppl_cols,

)

gold_list = df["Final_diagnosis1_PPL"].dropna().astype(float).tolist()

y_true_gold = np.r_[

np.ones(len(gold_list)),

np.zeros(len(incorrect_full_list))

]

scores_gold = -np.r_[gold_list, incorrect_full_list] # lower PPL = better

gold_res = bootstrap_auc_ci(y_true_gold, scores_gold, n_boot=2000, seed=42)

print(

f"[{model_name}] AUROC (Gold Standard vs {model_name} Incorrect DDx FULL): "

f"{gold_res['auc']:.4f} "

f"(95% CI {gold_res['ci_low']:.4f}-{gold_res['ci_high']:.4f})"

)

auc_rows.append({

"model": model_name,

"comparison": "Gold standard vs incorrect DDx FULL",

"auc": gold_res["auc"],

"ci_low": gold_res["ci_low"],

"ci_high": gold_res["ci_high"],

"n_positive": len(gold_list),

"n_negative": len(incorrect_full_list),

"n_boot_valid": gold_res["n_boot_valid"],

})

y_true_correct_full = np.r_[

np.ones(len(correct_list)),

np.zeros(len(incorrect_full_list))

]

scores_correct_full = -np.r_[correct_list, incorrect_full_list]

correct_full_res = bootstrap_auc_ci(

y_true_correct_full,

scores_correct_full,

n_boot=2000,

seed=42

)

print(

f"[{model_name}] AUROC ({model_name} Correct DDx vs {model_name} Incorrect DDx FULL): "

f"{correct_full_res['auc']:.4f} "

f"(95% CI {correct_full_res['ci_low']:.4f}-{correct_full_res['ci_high']:.4f})"

)

auc_rows.append({

"model": model_name,

"comparison": "Correct generated DDx vs incorrect DDx FULL",

"auc": correct_full_res["auc"],

"ci_low": correct_full_res["ci_low"],

"ci_high": correct_full_res["ci_high"],

"n_positive": len(correct_list),

"n_negative": len(incorrect_full_list),

"n_boot_valid": correct_full_res["n_boot_valid"],

})

y_true_correct_same = np.r_[

np.ones(len(correct_list)),

np.zeros(len(incorrect_same_case_list))

]

scores_correct_same = -np.r_[correct_list, incorrect_same_case_list]

correct_same_res = bootstrap_auc_ci(

y_true_correct_same,

scores_correct_same,

n_boot=2000,

seed=42

)

print(

f"[{model_name}] AUROC ({model_name} Correct DDx vs {model_name} Incorrect DDx SAME-CASE SUBSET): "

f"{correct_same_res['auc']:.4f} "

f"(95% CI {correct_same_res['ci_low']:.4f}-{correct_same_res['ci_high']:.4f})"

)

auc_rows.append({

"model": model_name,

"comparison": "Correct generated DDx vs incorrect DDx SAME-CASE SUBSET",

"auc": correct_same_res["auc"],

"ci_low": correct_same_res["ci_low"],

"ci_high": correct_same_res["ci_high"],

"n_positive": len(correct_list),

"n_negative": len(incorrect_same_case_list),

"n_boot_valid": correct_same_res["n_boot_valid"],

})

auc_summary_df = pd.DataFrame(auc_rows)

auc_summary_df

save_roc_plot_gold_vs_incorrect(gold_df, candidates, OUTDIR / 'roc_gold_vs_incorrect.png')

save_roc_plot_correct_vs_incorrect(candidates, OUTDIR / 'roc_correct_vs_incorrect_full.png')

save_distribution_plot(gold_df, candidates, OUTDIR / 'ppl_boxplot_logscale.png')

coverage.to_csv(OUTDIR / 'table1_coverage.csv', index=False)

summary.to_csv(OUTDIR / 'table2_ppl_summary.csv', index=False)

tests.to_csv(OUTDIR / 'statistical_tests.csv', index=False)

model_specific.to_csv(OUTDIR / 'model_specific_summary.csv', index=False)

auc_table.to_csv(OUTDIR / 'auc_summary.csv', index=False)

paired.to_csv(OUTDIR / 'paired_case_summary.csv', index=False)

candidates.to_csv(OUTDIR / 'candidate_level_data.csv', index=False)

gold_df.to_csv(OUTDIR / 'gold_standard_data.csv', index=False)

print('Saved outputs to:', OUTDIR.resolve())

expected = {

'LLaMA2': {'gold_vs_incorrect': 0.6669, 'same_case': 0.6423},

'Bard': {'gold_vs_incorrect': 0.7057, 'same_case': 0.6410},

'GPT': {'gold_vs_incorrect': 0.6259, 'same_case': 0.6932},

}

check_rows = []

for _, row in auc_table.loc[auc_table['model'].isin(expected.keys())].iterrows():

exp = expected[row['model']]

check_rows.append({

'model': row['model'],

'computed_gold_vs_incorrect': row['auc_gold_vs_incorrect_full'],

'expected_gold_vs_incorrect': exp['gold_vs_incorrect'],

'gold_match_4dp': round(row['auc_gold_vs_incorrect_full'], 4) == round(exp['gold_vs_incorrect'], 4),

'computed_same_case': row['auc_correct_vs_incorrect_same_case_subset'],

'expected_same_case': exp['same_case'],

'same_case_match_4dp': round(row['auc_correct_vs_incorrect_same_case_subset'], 4) == round(exp['same_case'], 4),

})

pd.DataFrame(check_rows)

auc_summary_df.to_csv(OUTDIR / "auc_summary_with_95ci.csv", index=False, encoding="cp932")

median_ci_df.to_csv(OUTDIR / "median_summary_with_95ci.csv", index=False, encoding="cp932")

********************************************************************************

#Adjusted, lexical-overlap, and case-level cluster-bootstrap analyses.

import os

import sys

import re

import argparse

from pathlib import Path

from collections import Counter

import numpy as np

import pandas as pd

from sklearn.metrics import roc_auc_score

import statsmodels.formula.api as smf

from scipy.stats import wilcoxon, chi2

DEFAULT_INPUT = (INPUT URL)

DEFAULT_OUTDIR = (OUTPUT URL)

BOOTSTRAPS = 1000

RANDOM_SEED = 20260623

def parse_args():

parser = argparse.ArgumentParser(

description="Adjusted, lexical-overlap, and cluster-bootstrap analyses for PPL data."

)

parser.add_argument("input_csv", nargs="?", default=DEFAULT_INPUT,

help="Path to input CSV file")

parser.add_argument("outdir", nargs="?", default=DEFAULT_OUTDIR,

help="Directory where output CSV/TXT files will be saved")

parser.add_argument("--bootstraps", type=int, default=BOOTSTRAPS,

help="Number of cluster-bootstrap resamples")

parser.add_argument("--seed", type=int, default=RANDOM_SEED,

help="Random seed")

args, unknown = parser.parse_known_args()

if str(args.input_csv) == "-f" or str(args.input_csv).endswith(".json"):

args.input_csv = DEFAULT_INPUT

if str(args.outdir).endswith(".json"):

args.outdir = DEFAULT_OUTDIR

args.input_csv = Path(os.path.expanduser(str(args.input_csv))).resolve()

args.outdir = Path(os.path.expanduser(str(args.outdir))).resolve()

return args

def read_csv_with_fallback(path: Path) -> pd.DataFrame:

encodings = ["cp932", "utf-8-sig", "utf-8", "shift_jis"]

last_error = None

for enc in encodings:

try:

return pd.read_csv(path, encoding=enc)

except UnicodeDecodeError as e:

last_error = e

raise last_error

def auc_safe(y, score, weight=None):

y = np.asarray(y)

score = np.asarray(score)

keep = np.isfinite(score)

if weight is not None:

weight = np.asarray(weight)

keep = keep & np.isfinite(weight) & (weight > 0)

y, score, weight = y[keep], score[keep], weight[keep]

else:

y, score = y[keep], score[keep]

if len(y) == 0 or len(np.unique(y)) < 2:

return np.nan

return float(roc_auc_score(y, score, sample_weight=weight))

def cluster_boot_auc_weighted(data, score_col, B, seed):

y = data["correct"].to_numpy()

score = data[score_col].to_numpy()

case_codes, case_ids = pd.factorize(data["case_id"])

n_cases = len(case_ids)

rng = np.random.default_rng(seed)

vals = []

for _ in range(B):

sampled = rng.integers(0, n_cases, size=n_cases)

counts = np.bincount(sampled, minlength=n_cases)

weights = counts[case_codes]

val = auc_safe(y, score, weights)

if not np.isnan(val):

vals.append(val)

if len(vals) == 0:

return np.nan, np.nan, 0

return float(np.percentile(vals, 2.5)), float(np.percentile(vals, 97.5)), len(vals)

def robust_result_cluster(fit, groups):

if hasattr(fit, "get_robustcov_results"):

return fit.get_robustcov_results(cov_type="cluster", groups=groups)

rob = fit._get_robustcov_results(cov_type="cluster", groups=groups)

return fit if rob is None else rob

def result_to_coef_table(result, param_index):

params = pd.Series(np.asarray(result.params), index=param_index)

conf_arr = np.asarray(result.conf_int())

pvals = pd.Series(np.asarray(result.pvalues), index=param_index)

return pd.DataFrame({

"term": param_index,

"coef": params.values,

"OR": np.exp(params.values),

"OR_CI_low": np.exp(conf_arr[:, 0]),

"OR_CI_high": np.exp(conf_arr[:, 1]),

"p_value": pvals.values,

})

def main():

args = parse_args()

input_csv = args.input_csv

outdir = args.outdir

B = args.bootstraps

seed = args.seed

if not input_csv.exists():

raise FileNotFoundError(f"Input CSV not found: {input_csv}")

outdir.mkdir(parents=True, exist_ok=True)

wide = read_csv_with_fallback(input_csv)

models = [

("LLaMA2", "llama2", "inclusion_llama2", "rank_llama2"),

("Bard", "Bard", "inclusion_Bard", "rank_Bard"),

("GPT-4", "GPT", "inclusion_GPT", "rank_GPT"),

]

records = []

for _, row in wide.iterrows():

case_id = int(row["No"])

context = str(row.get("final_case", "") or "")

case_words = len(re.findall(r"[A-Za-z0-9]+", context.lower()))

for model, suffix, inclusion_col, rank_col in models:

try:

correct_rank = int(row[rank_col])

except Exception:

correct_rank = 0

for k in range(1, 11):

dx = row.get(f"DDx{k}_{suffix}", "")

nll = pd.to_numeric(row.get(f"DDx{k}_{suffix}_avg_NLL", np.nan), errors="coerce")

ppl = pd.to_numeric(row.get(f"DDx{k}_{suffix}_PPL", np.nan), errors="coerce")

if pd.isna(dx) or str(dx).strip() == "" or pd.isna(nll) or pd.isna(ppl):

continue

records.append({

"case_id": case_id,

"model": model,

"candidate_rank_original": k,

"diagnosis": str(dx),

"avg_nll": float(nll),

"ppl": float(ppl),

"correct": 1 if correct_rank == k else 0,

"context": context,

"case_word_count": case_words,

})

long = pd.DataFrame(records)

if long.empty:

raise ValueError("No candidate records were created. Check CSV column names and suffixes.")

stopwords = set("""

a an and are as at be by for from in into is it its of on or the to with without due after before over under via vs versus

acute chronic disease syndrome diagnosis patient patients right left bilateral unilateral type caused associated induced secondary primary

possible probable suspected history result results finding findings positive negative normal abnormal clinical case report confirmed treated treatment

""".split())

def norm_text(s: str) -> str:

return re.sub(r"[^a-z0-9]+", " ", str(s).lower()).strip()

def content_tokens(s: str):

return [t for t in norm_text(s).split() if len(t) > 1 and t not in stopwords]

long["dx_norm"] = long["diagnosis"].map(norm_text)

long["context_norm"] = long["context"].map(norm_text)

long["dx_token_count_raw"] = long["diagnosis"].map(

lambda x: len(re.findall(r"[A-Za-z0-9]+", str(x).lower()))

)

long["dx_char_count"] = long["diagnosis"].map(lambda x: len(str(x)))

long["dx_tokens_content"] = long["diagnosis"].map(content_tokens)

all_tokens = []

for toks in long["dx_tokens_content"]:

all_tokens.extend(set(toks))

freq = Counter(all_tokens)

N = len(long)

long["mean_log_token_df"] = long["dx_tokens_content"].map(

lambda toks: float(np.mean([np.log1p(freq[t]) for t in set(toks)])) if set(toks) else 0.0

)

long["mean_token_idf"] = long["dx_tokens_content"].map(

lambda toks: float(np.mean([np.log((N + 1) / (freq[t] + 1)) + 1 for t in set(toks)])) if set(toks) else 0.0

)

context_sets = [set(s.split()) for s in long["context_norm"]]

overlap_fraction = []

overlap_count = []

exact_phrase = []

for toks, dx_norm, context_norm, ctx_set in zip(

long["dx_tokens_content"], long["dx_norm"], long["context_norm"], context_sets

):

toks = set(toks)

n_overlap = len(toks & ctx_set) if toks else 0

overlap_count.append(n_overlap)

overlap_fraction.append(n_overlap / len(toks) if toks else 0.0)

exact_phrase.append(int(dx_norm != "" and (" " + dx_norm + " ") in (" " + context_norm + " ")))

long["overlap_count"] = overlap_count

long["overlap_fraction"] = overlap_fraction

long["any_overlap"] = (long["overlap_count"] > 0).astype(int)

long["exact_phrase_in_context"] = exact_phrase

long["neg_avg_nll"] = -long["avg_nll"] # higher means more compatible/lower perplexity

long["log_ppl"] = np.log(long["ppl"])

long["case_word_count_1000"] = long["case_word_count"] / 1000.0

counts_rows = []

datasets = [("Pooled", long)] + [(m, long[long["model"] == m]) for m in ["LLaMA2", "Bard", "GPT-4"]]

for name, d in datasets:

counts_rows.append({

"dataset": name,

"candidates": len(d),

"correct": int(d["correct"].sum()),

"incorrect": int((1 - d["correct"]).sum()),

"cases": int(d["case_id"].nunique()),

})

counts = pd.DataFrame(counts_rows)

auc_rows = []

score_specs = [

("neg_avg_nll", "Conditional perplexity / -avgNLL"),

("overlap_fraction", "Lexical overlap fraction"),

("any_overlap", "Any lexical overlap"),

("exact_phrase_in_context", "Exact phrase in context"),

]

for name, d in datasets:

for score_col, label in score_specs:

auc = auc_safe(d["correct"], d[score_col])

lo, hi, n_boot = cluster_boot_auc_weighted(

d, score_col, B=B, seed=seed + len(name) + len(score_col)

)

auc_rows.append({

"dataset": name,

"score": label,

"AUC": auc,

"cluster_CI_low": lo,

"cluster_CI_high": hi,

"n_boot": n_boot,

})

auc_table = pd.DataFrame(auc_rows)

formula = (

"correct ~ neg_avg_nll + dx_token_count_raw + mean_log_token_df + "

"overlap_fraction + exact_phrase_in_context + case_word_count_1000 + C(model)"

)

fit = smf.logit(formula, data=long).fit(disp=False, maxiter=200)

rob = robust_result_cluster(fit, groups=long["case_id"])

coef_table = result_to_coef_table(rob, fit.params.index)

long["adjusted_pred"] = fit.predict(long)

adjusted_auc = auc_safe(long["correct"], long["adjusted_pred"])

adj_lo, adj_hi, adj_n_boot = cluster_boot_auc_weighted(

long, "adjusted_pred", B=B, seed=seed + 99

)

model_formulas = {

"Lexical-only": (

"correct ~ dx_token_count_raw + mean_log_token_df + overlap_fraction + "

"exact_phrase_in_context + case_word_count_1000 + C(model)"

),

"PPL-only": "correct ~ neg_avg_nll + C(model)",

"PPL + lexical adjusted": formula,

}

model_auc_rows = []

fitted = {}

for label, frm in model_formulas.items():

f = smf.logit(frm, data=long).fit(disp=False, maxiter=200)

fitted[label] = f

pred_col = "pred_" + re.sub(r"[^A-Za-z0-9]+", "_", label).strip("_").lower()

long[pred_col] = f.predict(long)

auc = auc_safe(long["correct"], long[pred_col])

lo, hi, _ = cluster_boot_auc_weighted(

long, pred_col, B=B, seed=seed + 200 + len(label)

)

model_auc_rows.append({

"model": label,

"AUC": auc,

"cluster_CI_low": lo,

"cluster_CI_high": hi,

})

model_auc = pd.DataFrame(model_auc_rows)

reduced = fitted["Lexical-only"]

lr_stat = 2 * (fit.llf - reduced.llf)

df_diff = int(fit.df_model - reduced.df_model)

lr_p = chi2.sf(lr_stat, df_diff)

wc_rows = []

for model in ["LLaMA2", "Bard", "GPT-4"]:

dm = long[long["model"] == model]

pairs = []

for case_id, g in dm.groupby("case_id"):

if g["correct"].sum() != 1:

continue

correct_ppl = float(g.loc[g["correct"] == 1, "ppl"].iloc[0])

inc = g.loc[g["correct"] == 0, "ppl"]

pairs.append((case_id, correct_ppl, float(inc.min()), float(inc.median()), float(inc.mean())))

pair_df = pd.DataFrame(

pairs,

columns=["case_id", "correct_ppl", "best_incorrect_ppl", "median_incorrect_ppl", "mean_incorrect_ppl"],

)

for comparator in ["best_incorrect_ppl", "median_incorrect_ppl", "mean_incorrect_ppl"]:

p = wilcoxon(pair_df["correct_ppl"], pair_df[comparator], alternative="less").pvalue

wc_rows.append({

"model": model,

"paired_cases": len(pair_df),

"comparator": comparator,

"correct_lower_n": int((pair_df["correct_ppl"] < pair_df[comparator]).sum()),

"correct_lower_pct": 100 * float((pair_df["correct_ppl"] < pair_df[comparator]).mean()),

"wilcoxon_p_less": p,

"median_correct_ppl": float(pair_df["correct_ppl"].median()),

"median_comparator_ppl": float(pair_df[comparator].median()),

})

within_case = pd.DataFrame(wc_rows)

feature_summary = long.groupby("correct").agg(

n=("correct", "size"),

avg_NLL_median=("avg_nll", "median"),

PPL_median=("ppl", "median"),

dx_token_count_median=("dx_token_count_raw", "median"),

overlap_fraction_median=("overlap_fraction", "median"),

any_overlap_pct=("any_overlap", lambda x: 100 * np.mean(x)),

exact_phrase_pct=("exact_phrase_in_context", lambda x: 100 * np.mean(x)),

mean_log_token_df_median=("mean_log_token_df", "median"),

).reset_index()

feature_summary["correct"] = feature_summary["correct"].map({0: "Incorrect", 1: "Correct"})

export_long = long.drop(columns=["context", "context_norm", "dx_tokens_content"])

export_long.to_csv(outdir / "candidate_level_with_lexical_features.csv", index=False)

counts.to_csv(outdir / "counts.csv", index=False)

auc_table.to_csv(outdir / "cluster_bootstrap_auc.csv", index=False)

coef_table.to_csv(outdir / "adjusted_logistic_cluster_robust_coefficients.csv", index=False)

model_auc.to_csv(outdir / "prediction_model_auc.csv", index=False)

within_case.to_csv(outdir / "within_case_sensitivity.csv", index=False)

feature_summary.to_csv(outdir / "feature_summary_by_correctness.csv", index=False)

with open(outdir / "summary.txt", "w", encoding="utf-8") as f:

f.write("Input CSV: " + str(input_csv) + "\n")

f.write("Output directory: " + str(outdir) + "\n")

f.write(f"Bootstraps: {B}\n\n")

f.write("Counts\n" + counts.to_string(index=False) + "\n\n")

f.write("AUCs\n" + auc_table.to_string(index=False, float_format=lambda x: f"{x:.3f}") + "\n\n")

f.write("Feature summary\n" + feature_summary.to_string(index=False, float_format=lambda x: f"{x:.3f}") + "\n\n")

f.write("Adjusted logistic regression\n" + coef_table.to_string(index=False, float_format=lambda x: f"{x:.4g}") + "\n")

f.write(f"\nAdjusted prediction AUC: {adjusted_auc:.3f} ")

f.write(f"(cluster bootstrap 95% CI {adj_lo:.3f}-{adj_hi:.3f}; n_boot={adj_n_boot})\n\n")

f.write("Prediction model AUCs\n" + model_auc.to_string(index=False, float_format=lambda x: f"{x:.3f}") + "\n")

f.write(f"\nLR test adding PPL term to lexical-only model: chi2={lr_stat:.2f}, df={df_diff}, P={lr_p:.3g}\n\n")

f.write("Within-case sensitivity\n" + within_case.to_string(index=False, float_format=lambda x: f"{x:.4g}") + "\n")

print("Wrote outputs to", outdir)

print(counts.to_string(index=False))

print(auc_table.to_string(index=False, float_format=lambda x: f"{x:.3f}"))

print(coef_table.to_string(index=False, float_format=lambda x: f"{x:.4g}"))

print(model_auc.to_string(index=False, float_format=lambda x: f"{x:.3f}"))

print(f"LR test adding PPL term to lexical-only model: chi2={lr_stat:.2f}, df={df_diff}, P={lr_p:.3g}")

if __name__ == "__main__":

main()
