# Supplementary material for: Conditional Perplexity Scoring for Large Language Model−Generated Differential Diagnoses in Case Reports: Preliminary Computational Evaluation
Source: JMIR Form Res. 2026 Sep 9;10:e98819. doi: 10.2196/98819 (PMC13557325; doi:10.2196/98819)
Supplement: Supplementary file 3 [file formative-v10-e98819-s003.docx]

# Multimedia Appendix 3

Supplemental sensitivity analyses addressing lexical overlap, adjusted models, case-level cluster-bootstrap AUCs, and within-case comparator analyses

Purpose. These post hoc analyses were conducted in response to reviewer concerns that conditional perplexity may reflect diagnosis-string length, token/lexical frequency, lexical overlap with the case narrative, or clustering of candidates within cases. The analyses are exploratory and should be interpreted as robustness checks rather than definitive validation of clinical reasoning.

Analytic definitions. Conditional perplexity score (PPL) was analyzed using average negative log-likelihood (avgNLL) and -avgNLL, where larger -avgNLL corresponds to lower perplexity and greater model predictability. Lexical features included diagnosis-string token count, character count, mean log token document frequency within the candidate corpus, token-overlap fraction between the diagnosis string and pre-diagnostic case narrative, any token overlap, exact phrase occurrence in the narrative, and case word count. Confidence intervals for AUCs were estimated using a case-level cluster bootstrap with 1000 resamples.

Supplemental Table 3-1. Candidate-level analytic counts.

| **Dataset** | Candidates | Correct diagnosis strings | Incorrect diagnosis strings |
| --- | --- | --- | --- |
| **Pooled** |  |  |  |
|  | 11698 | 823 | 10875 |
| **LLaMA2** |  |  |  |
|  | 3896 | 214 | 3682 |
| **Bard** |  |  |  |
|  | 3899 | 269 | 3630 |
| **GPT-4** |  |  |  |
|  | 3903 | 340 | 3563 |

Supplemental Table 3-2. Case-level cluster-bootstrap AUC for conditional perplexity and lexical baselines.

| **Dataset** | **Score** | AUC | 95% cluster-bootstrap CI | Bootstrap resamples |
| --- | --- | --- | --- | --- |
| **Pooled** | **Conditional perplexity / -avgNLL** |  |  |  |
|  |  | 0.666 | 0.644-0.689 | 1000 |
| **Pooled** | **Lexical overlap fraction** |  |  |  |
|  |  | 0.570 | 0.545-0.596 | 1000 |
| **Pooled** | **Any lexical overlap** |  |  |  |
|  |  | 0.567 | 0.542-0.589 | 1000 |
| **Pooled** | **Exact phrase in context** |  |  |  |
|  |  | 0.500 | 0.485-0.515 | 1000 |
| **LLaMA2** | **Conditional perplexity / -avgNLL** |  |  |  |
|  |  | 0.662 | 0.625-0.698 | 1000 |
| **LLaMA2** | **Lexical overlap fraction** |  |  |  |
|  |  | 0.586 | 0.550-0.621 | 1000 |
| **LLaMA2** | **Any lexical overlap** |  |  |  |
|  |  | 0.578 | 0.545-0.608 | 1000 |
| **LLaMA2** | **Exact phrase in context** |  |  |  |
|  |  | 0.502 | 0.476-0.527 | 1000 |
| **Bard** | **Conditional perplexity / -avgNLL** |  |  |  |
|  |  | 0.648 | 0.617-0.681 | 1000 |
| **Bard** | **Lexical overlap fraction** |  |  |  |
|  |  | 0.573 | 0.542-0.605 | 1000 |
| **Bard** | **Any lexical overlap** |  |  |  |
|  |  | 0.571 | 0.540-0.599 | 1000 |
| **Bard** | **Exact phrase in context** |  |  |  |
|  |  | 0.508 | 0.483-0.532 | 1000 |
| **GPT-4** | **Conditional perplexity / -avgNLL** |  |  |  |
|  |  | 0.678 | 0.652-0.705 | 1000 |
| **GPT-4** | **Lexical overlap fraction** |  |  |  |
|  |  | 0.575 | 0.547-0.607 | 1000 |
| **GPT-4** | **Any lexical overlap** |  |  |  |
|  |  | 0.565 | 0.540-0.593 | 1000 |
| **GPT-4** | **Exact phrase in context** |  |  |  |
|  |  | 0.506 | 0.493-0.520 | 1000 |

Supplemental Table 3-3. Lexical-feature summary by physician-verified correctness.

| **Group** | n | Median avgNLL | Median PPL | Median diagnosis tokens | Median overlap fraction | Any overlap, % | Exact phrase, % | Median mean log token DF |
| --- | --- | --- | --- | --- | --- | --- | --- | --- |
| **Correct** |  |  |  |  |  |  |  |  |
|  | 823 | 3.768 | 43.308 | 3.000 | 0.500 | 61.939 | 14.545 | 3.061 |
| **Incorrect** |  |  |  |  |  |  |  |  |
|  | 10875 | 4.892 | 133.266 | 2.000 | 0.000 | 48.570 | 14.605 | 3.333 |

Supplemental Table 3-4. Adjusted logistic regression predicting physician-verified correctness with case-clustered standard errors.

| **Variable** | OR | 95% CI | *P* value |
| --- | --- | --- | --- |
| **Intercept** |  |  |  |
|  | 0.53 | 0.33-0.84 | .007 |
| **Source model: GPT-4 vs Bard** |  |  |  |
|  | 1.16 | 1.06-1.27 | .002 |
| **Source model: LLaMA2 vs Bard** |  |  |  |
|  | 0.73 | 0.65-0.81 | <.001 |
| **-average NLL** |  |  |  |
|  | 1.36 | 1.27-1.46 | <.001 |
| **Diagnosis token count** |  |  |  |
|  | 0.93 | 0.88-0.98 | .008 |
| **Mean log token document frequency proxy** |  |  |  |
|  | 0.84 | 0.77-0.92 | <.001 |
| **Lexical-overlap fraction** |  |  |  |
|  | 2.80 | 2.08-3.77 | <.001 |
| **Exact phrase in context** |  |  |  |
|  | 0.48 | 0.35-0.66 | <.001 |
| **Case word count, per 1000 words** |  |  |  |
|  | 1.00 | 0.75-1.32 | .988 |

Supplemental Table 3-5. Prediction-model AUCs for lexical-only, perplexity-only, and combined models.

| **Model** | AUC | 95% cluster-bootstrap CI |
| --- | --- | --- |
| **Lexical-only** |  |  |
|  | 0.639 | 0.618-0.662 |
| **PPL-only** |  |  |
|  | 0.671 | 0.649-0.694 |
| **PPL + lexical adjusted** |  |  |
|  | 0.694 | 0.672-0.716 |

Supplemental Table 3-6. Per-source-model adjusted association and adjusted-model AUC.

| **Source model** | OR for -average NLL | 95% CI for OR | *P* value | Adjusted-model AUC | 95% cluster-bootstrap CI for AUC |
| --- | --- | --- | --- | --- | --- |
| **LLaMA2** |  |  |  |  |  |
|  | 1.37 | 1.23-1.52 | <.001 | 0.684 | 0.649-0.721 |
| **Bard** |  |  |  |  |  |
|  | 1.31 | 1.20-1.43 | <.001 | 0.674 | 0.644-0.704 |
| **GPT-4** |  |  |  |  |  |
|  | 1.46 | 1.32-1.61 | <.001 | 0.720 | 0.690-0.747 |

Supplemental Table 3-7. Within-case comparator sensitivity analyses.

| **Source model** | Paired cases | Comparator | Correct lower, n (%) | Wilcoxon *P* value | Median correct PPL | Median comparator PPL |
| --- | --- | --- | --- | --- | --- | --- |
| **LLaMA2** |  |  |  |  |  |  |
|  | 214 | Lowest-perplexity incorrect diagnosis | 54 (25.2) | 1.000 | 45.2 | 14.0 |
| **LLaMA2** |  |  |  |  |  |  |
|  | 214 | Median incorrect diagnosis | 150 (70.1) | <.001 | 45.2 | 133.3 |
| **LLaMA2** |  |  |  |  |  |  |
|  | 214 | Mean incorrect diagnosis | 195 (91.1) | <.001 | 45.2 | 872.2 |
| **Bard** |  |  |  |  |  |  |
|  | 269 | Lowest-perplexity incorrect diagnosis | 64 (23.8) | 1.000 | 55.9 | 17.9 |
| **Bard** |  |  |  |  |  |  |
|  | 269 | Median incorrect diagnosis | 183 (68.0) | <.001 | 55.9 | 175.3 |
| **Bard** |  |  |  |  |  |  |
|  | 269 | Mean incorrect diagnosis | 237 (88.1) | <.001 | 55.9 | 1701.3 |
| **GPT-4** |  |  |  |  |  |  |
|  | 340 | Lowest-perplexity incorrect diagnosis | 82 (24.1) | 1.000 | 30.7 | 10.7 |
| **GPT-4** |  |  |  |  |  |  |
|  | 340 | Median incorrect diagnosis | 252 (74.1) | <.001 | 30.7 | 105.4 |
| **GPT-4** |  |  |  |  |  |  |
|  | 340 | Mean incorrect diagnosis | 304 (89.4) | <.001 | 30.7 | 686.3 |

Interpretation

Lexical overlap alone produced weaker discrimination than conditional perplexity, and exact phrase occurrence in the narrative was near chance as a classifier. Lower avgNLL remained associated with physician-verified correctness after adjustment for multiple lexical features and source model. However, the within-case best-incorrect comparison shows that conditional perplexity is not sufficient as a standalone reranking rule: the correct diagnosis frequently did not have the lowest perplexity among all candidates in a list.

Remaining limitations

These analyses used a token-frequency proxy derived from the candidate-diagnosis corpus, not the scorer model's pretraining distribution or unconditional token probabilities. The current dataset also did not include diagnosis-term masking or mismatched-context rescoring. Those analyses require additional model inference and should be considered priorities for future validation.
